# Optimizing vaccination strategies under uncertainty to prevent epidemics

**DOI:** 10.3389/fpubh.2026.1761731

**Published:** 2026-02-24

**Authors:** Lewis Ntaimo, Mjumo Mzyece, David R. Katerere

**Affiliations:** 1 Wm Michael Barnes Department of Industrial & Systems Engineering, Texas A&M University, College Station, TX, United States; 2 Business and Economics Department, Northwestern College, Orange City, IA, United States; 3 Department of Pharmaceutical Sciences, Tshwane University of Technology, Pretoria, South Africa

**Keywords:** COVID-19, epidemics, infectious diseases, integrated chance constraints, optimization, public health, stochastic programming, vaccination strategies

## Abstract

To be prepared for future infectious disease epidemics, this paper considers a data-driven approach for determining optimal vaccination strategies for multi-community settings with heterogeneous populations under uncertain social mixing, disease transmission, and vaccine efficacy. Specifically, we derive an integrated chance constraints stochastic programming (ICC-SP) disease spread model for optimal vaccination strategies to prevent epidemics by keeping the uncertain post-vaccination reproduction number below one at a specified level of risk. A vaccination strategy specifies the proportion of individuals in a given household-type and age-group to vaccinate and the intervention level needed to bound the expected excess of the reproduction number above one by an acceptable level of reliability (or risk). The ICC-SP model is data-driven and uses readily available data on census demographics, age-related disease susceptibility and infectivity, virus variants, and vaccine efficacy defined in three ways: vaccination in terms of effectiveness against infection, symptomatic cases, and hospitalization. This data-driven approach incorporates the decision-maker's level of risk to enable public health policy what-if analyses for future epidemics. A case study using the ICC-SP approach based on COVID-19 data was conducted, and the results of the study provide several insights. The study shows, for example, that to control disease outbreaks vaccination strategies must be combined with a specific intervention level. The proportion of the population to vaccinate to prevent epidemics depends on the criterion of vaccine efficacy used and decreases with increasing intervention level. The study also shows that optimal vaccination strategies prioritize the vaccination of specific households and age groups with high combined levels of relative susceptibility and infectivity.

## 1 Introduction

In this work, we derive a data-driven integrated chance constraints stochastic optimization approach for finding optimal vaccination strategies under uncertainty to prevent epidemics in a multi-community setting. This new approach involves a stochastic optimization model of disease spread for multi-community settings. The model uses readily available population demographic data together with uncertain disease spread and vaccine efficacy data. This work is motivated by COVID-19 which has continued to spread globally since early 2020 resulting in extremely high mortality, morbidity, and hospitalizations. We now have new variants with uncertain disease transmission characteristics (1), and as can be seen in Table 1, the disease is still active worldwide but no longer at the pandemic scale. Both South Africa and the U.S. have active cases. We believe that it is only a matter of time before another epidemic or pandemic occurs (2). Therefore, it is imperative to be prepared and continue developing new data-driven infectious disease spread models and methods that will aid decision making during future epidemics.

**Table 1:** COVID-19 cases by April 2024 (41).

	**Total**	**Total**	**Total**	**Total cases/**	**Total deaths/**	
**Country**	**Cases**	**Deaths**	**Recovered**	**1M population**	**1M population**	**Population**
Worldwide	704,753,890	7,010,681	675,619,811	90,413	899.4	
South Africa	4,076,463	102,595	3,912,506	67,095	1,689	60,756,135
USA	111,820,082	1,219,487	109,814,428	333,985	3,642	334,805,269

The negative impact of the pandemic worldwide is still felt today, as it has changed our daily lives, not to mention the socio-economic impacts. To fight the pandemic, both pharmaceutical and non-pharmaceutical interventions were implemented and came at high social and economic cost. These costs are estimated to exceed those associated with conventional recessions and modern wars by far (3). It cost more than $16 trillion by 2021 to fight the disease in the U.S. alone (3). In South Africa, for the 2020/21 fiscal year from April 2020 to March 2021, the cost was estimated at R142 billion (about $8.67 billion) of which R21.544 billion (about $1.32 billion) was spent directly on health care services (4). Pharmaceutical interventions included vaccinations and therapies, while non-pharmaceutical interventions included quarantines, lockdowns, and mask mandates.

Clinical trials are usually conducted to estimate the effectiveness of a vaccine or *vaccine efficacy* (VE), which is the percentage reduction in disease among the vaccinated. There are several ways vaccine efficacy and effectiveness are determined, e.g., vaccine efficacy against *infection, severity of symptoms, hospitalization*, or *mortality*. The reported vaccine efficacy values vary significantly, with some vaccines being relatively effective against certain virus variants (5). Vaccine efficacy can also be measured by how it modulates severity of symptoms, reduction in hospitalization as well as the length of hospitalization, and reduction in morbidity. Each of the virus variants has its own spread characteristics and how it infects and affects an individual based on age in terms of their resulting *susceptibility* and *infectivity*. Susceptibility is the ability to become infected if in *close contact* with an infected individual, whereas infectivity is the ability of an infected individual to spread the disease to others. A *close contact* is when a susceptible individual is within the proximity of an infected individual and may possibly transmit the disease. Vaccine efficacy and an individual's susceptibility, infectivity, and vaccine efficacy are all uncertain. Consequently, this poses challenges in modeling optimal vaccine allocation strategies toward preventing and controlling infectious diseases. This paper makes advances by incorporating uncertainty in close contact within and outside the household, susceptibility, infectivity, and vaccine efficacy into the optimization model.

Non-pharmaceutical interventions during the peak of the pandemic included mask mandates, social distancing, gathering restrictions, quarantining, contact tracing, lockdowns, and border closures. Beginning in early 2020, several countries developed monitoring and alert systems. For example, South Africa enacted five intervention levels, *Alert Levels 1* to *5* (6). The lowest alert level (*Level 1*) allows for most normal activities with precautions and health guidelines followed at all times, while the highest alert level (*Level 5*) imposes drastic measures such as prohibiting mass gatherings, to contain the spread of the virus and reduce disease burden. Non-pharmaceutical interventions affect the spread of an infectious disease outside the household, which is uncertain at best and is measured in terms of close contact rate.

An important concept in epidemiology to determine whether an epidemic will continue or not is the *basic reproduction number* *R*_0_, which is the average number of secondary infections produced by a typical case of an infection in a population (7, 8). This means that during an epidemic, *R*_0_>1. However, what is actually measured during an epidemic at a given time *t* is the *effective* reproduction number, *R*(*t*) (9, 10). During a vaccination campaign, the goal is to have the vaccine induce herd immunity in the population, and in this case, the *post-vaccination* reproduction number of infected households *R*_*HV*_, is measured. To prevent epidemics *R*_*HV*_ ≤ 1, and the objective is to optimize vaccination coverage, i.e., to determine the minimum proportion of the population to vaccinate (11). The challenge toward achieving this lies in characterizing *R*_*HV*_ since it is a random variable and involves several parameters (e.g., vaccine efficacy, susceptibility, and infectivity) all of which are uncertain.

We define an *optimal vaccination strategy* as the fraction (or percentage) of the population in each age group to vaccinate using the minimum number of vaccines to prevent epidemics at a specified reliability level for a given community. Therefore, we take a mathematical programming approach that integrates a disease spread model based on (11) and stochastic programming (12–14). Specifically, we devise an integrated chance constraints stochastic programming (ICC-SP) model that extends a previous model based on chance constraints (15). The idea of using ICC-SP in the epidemiology setting was first introduced by (16) to enable determining data-driven vaccination strategies under a stochastic post vaccination reproduction number. This work continues along the same vein by: (a) incorporating intervention levels into the model as binary decisions; (b) incorporating three types of vaccine efficacy, which do not include mortality: vaccination effectiveness against infection (VEI), vaccination effectiveness against symptomatic cases (VES), and vaccination effectiveness against hospitalization (VEH); and (c) applying the model to historical data for the South Africa setting.

We give a summary of closely related work on optimal vaccination strategies in Table 2. The table gives the citation, the model type (deterministic, simulation, and stochastic), and whether the model includes the following factors: households, age-specific heterogeneity in disease susceptibility and infectivity, vaccine efficacy type (VEI, VES, and VEH) used, and intervention levels. The symbol ✓ in the table means that the model includes the factor, while the symbol X means that the model does not include that factor. The main contributions of this work to the literature on vaccine allocation models include a new data-driven ICC-SP model for determining optimal vaccination strategies for multi-community settings, a computational study illustrating the application of the model using COVID-19 data for the South Africa multi-community setting, and the insights drawn from the study. The ICC-SP model provides an advance over previous related models by incorporating VEI, VES, and VEH, and intervention levels. Public health decision-makers need such data-driven models to aid in determining optimal vaccination strategies to prevent epidemics. The data-driven ICC-SP model is versatile in the sense that it uses readily available census demographic data, available disease spread data, and social interaction data for the communities under study. Thus, the ICC-SP model can be applied to any multi-community setting where the aforementioned data are available. Besides determining optimal vaccination strategies for each community, the model has a unique feature, namely selecting the optimal intervention level to prevent epidemics. This is especially important for public health authorities that need data-driven models to decide the appropriate level of intervention for each community during epidemics.

**Table 2:** Closely related work on optimal vaccination strategies.

		**Model factors**					
			**Age-specific**	**Vaccine efficacy**			**Intervention**
**Paper**	**Model type**	**Household**	**Heterogeneity**	**VEI**	**VES**	**VEH**	**Levels**
(42)	Deterministic	✓	X	✓	X	X	X
(39)	Stochastic (CC)	✓	X	✓	X	X	X
(43)	Deterministic	X	X	✓	X	X	X
(44)	Deterministic	X	✓	✓	X	X	X
(45)	Simulation	X	✓	✓	✓	✓	X
(15)	Stochastic (CC)	✓	✓	✓	X	X	X
(16)	Stochastic (ICC)	✓	✓	✓	X	X	X
This work	Stochastic (ICC)	✓	✓	✓	✓	✓	✓

CC, Chance Constraints; ICC, Integrated Chance Constraints; VEI, Vaccination Effectiveness Against Infection; VES, Vaccination Effectiveness Against Symptoms; VEH, Vaccination Effectiveness Against Hospitalization.

## 2 Materials and methods

**Figure 1:**
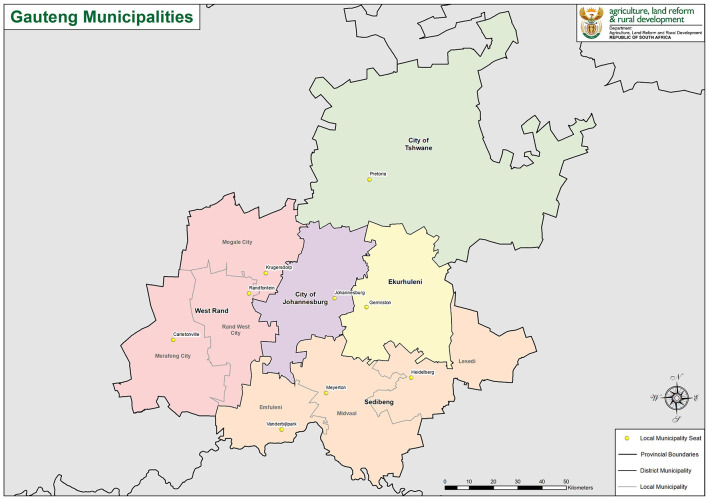
Map showing the five municipalities (Ekurhuleni, Johannesburg, Sedibeng, Tshwane, and West Rand) in Gauteng Province, South Africa, used in the case study (https://en.wikipedia.org/wiki/List_of_municipalities_in_Gauteng).

In this section, we describe the materials and methods used in our study. We start with the description of the model data in the next subsection and then present our data-driven multi-community stochastic model of disease spread in the subsequent subsection. We apply our methodology in a case study that involves census demographic and COVID-19 data for the South Africa setting encompassing five contiguous municipalities in Gauteng province as shown in Figure 1: City of Johannesburg, Ekurhuleni, City of Tshwane, Sedibeng (comprising Emfuleni, Midvaal, and Lesedi), and West Rand (comprising Merafong City, Rand West City, and Mogale City). Since the first case of COVID-19 was reported in South Africa in March 2020, the country experienced several waves of COVID-19 infections, first due to the delta variant, then in November 2021 South Africa was one of the first countries to report the presence of the Omicron variant (17). However, data on the effective reproduction number for each municipality during this period are not available (18).

### 2.1 Model data

Model data include census demographic data, outside transmission rate, inside household transmission rate, vaccine efficacy, relative susceptibility, relative infectivity, and model reliability level. These data are described in detail in the following subheadings.

#### 2.1.1 Demographic data

We consider actual census demographic data for all the communities (municipalities) included in the study area. Each community is defined by the discrete distribution of its different household types. For a homogeneous model, a household type is determined by the number of members in a household following a univariate discrete distribution. In a heterogeneous model, such as the one we consider, the household type is a multivariate discrete distribution defined by the size of the household and the number of household members in each age group *A, B, C*, and *D*. We consider maximum household size of 10. The four age groups *A, B, C*, and *D* are defined as follows: *A*−*age* ≤ 19*yrs*., *B*−20 ≤ *age* ≤ 39*yrs*., *C*−40 ≤ *age* ≤ 64*yrs*., and *D*−*age*≥65*yrs*. These age groups are defined based on the variation of the effect of COVID-19 on different age groups. The household size distribution data for each of the municipalities in Gauteng province were downloaded from the reports in the Census 2022 Products section of the census website for the year 2022 (19). The population data for each municipality are as follows (20): Ekurhuleni has a population of 4,024,285 and a population density of 2,038 people per square km; Johannesburg has a population of 5,079,469 and a population density of 3,088 people per square km; Sedibeng has a population of 1,125,282 and a population density of 270 people per square km; Tshwane has a population of 3,832,516 and a population density of 609 people per square km; and West Rand has a population of 1,007,757 and a population density of 247 people per square km. Clearly, the population density varies significantly across the municipalities. The demographic distribution data we constructed and utilized for our case study is made available in Supplementary material.

#### 2.1.2 Household transmission rate

The household transmission rate model parameter $b\left(\tilde{\omega }\right)$ is a measure of how many people in a household become infected after being exposed to an infected person. It captures the transmissibility of an infection within a household in a community *c*, where $0\leq b\left({\tilde{\omega }}_{c}\right)\leq 1$. The extreme value of $b\left({\tilde{\omega }}_{c}\right)=0$ is equivalent to no disease transmission within a household while the value of $b\left({\tilde{\omega }}_{c}\right)=1$ implies that all members of the household become infected (11). A meta-analysis of household transmission studies conducted between March and June 2020 found an overall secondary attack rate of 15.5% (21). However, data on household transmission rates for 2021 and 2022 is not available. The household transmission rate is analogous to household secondary attack rate, which is the probability that an infected individual will transmit the disease to a susceptible individual (22).

#### 2.1.3 Outside household close contacts

An important parameter in the model is the outside household close contacts $m\left({\tilde{\omega }}_{c}\right)$. It is defined as the average number of close contacts that an infected individual makes with persons from other households in the course of his/her infectious period. Recall that *close contact* means that the interaction between the infected individual is sufficient for transmitting the disease to the susceptible individual. The parameter $m\left({\tilde{\omega }}_{c}\right)$ is a random variable due to differences in human interactions under the impact of various mitigation measures, social conditions, and demographics of a community. The South African government enacted five COVID-19 intervention levels (6) which are summarized as follows:

- *Level 1:* Most normal activity can resume with precautions and health guidelines followed at all times. Population prepared for an increase in alert levels if necessary.
- *Level 2:* Physical distancing and restrictions on leisure and social activities to prevent a resurgence of the virus.
- *Level 3:* Restrictions on many activities, including at workplaces and socially, to address a high risk of transmission.
- *Level 4:* Extreme precautions to limit community transmission and outbreaks while allowing some activity to resume.
- *Level 5:* Drastic measures to contain the spread of the virus and save lives.

Using standard deterministic compartmental SEIR (Susceptible, Exposed, Infected, and Recovered) modeling, an effective contact rate of 1.30 (95% credible interval [Crl] 1.21–1.39) per day, incubation period of 3.21 days (95% Crl 3.04–3.44 days), infectious period of 2.27 days (95% Crl 2.04–2.74 days), and *R*_0_ of 2.95 (95% Crl 2.83–3.33) were reported before the *lockdown* (23). The lockdown resulted in an 80.3% reduction in effective contacts. To estimate the distribution of $m\left(\tilde{\omega_{c}}\right)$ in Gauteng province, we collected and used historical time series data for the effective reproduction number *R*_*t*_ from (24). For each municipality, we generated discrete distributions for relative $m\left({\tilde{\omega }}_{c}\right)$ for each level ℓ∈*L*, which we use in our computational study. These data are included in Supplementary material.

#### 2.1.4 Vaccine efficacy

Vaccine efficacy quantifies how well vaccination protects individuals from health outcomes such as infection, symptomatic illness, hospitalization, and death. Typically, it is measured by comparing the frequency of these health outcomes in vaccinated vs. unvaccinated people. The vaccine efficacy model parameter $\epsilon_{k}\left({\tilde{\omega }}_{c}\right)$ captures the efficacy of the vaccine. The aim of our study is to determine optimal vaccination strategies under three different *vaccine efficacy* criteria: VEI, VES, and VEH. The vaccines available in South Africa during the COVID-19 pandemic at different times include AstraZeneca-University of Oxford (also known as CoviShield), Johnson & Johnson (J&J), and Pfizer (Comirnaty). These vaccines have reported vaccine efficacy that ranged from 60% to 85%. The vaccine efficacy varies as a result of variability in real-world conditions, such as how the vaccine is transported, how the vaccine is administered, and the medical condition of vaccinated persons. Other important factors that affect the efficacy of these vaccines include the emergence of new evasive or resistant variants of the virus and the age of the person receiving the vaccine. For example, the SARS-CoV-2 virus has mutated into new variants including *Alpha, Beta, Gamma, Delta, Epsilon, Eta, Iota, Kappa, Omicron, Zeta*, and *Mu*.

While the Oxford-AstraZeneca vaccine was the first to be available in South Africa and initially used against the 501.V2 variant, by February 2021 its use was curtailed due to low efficacy. It was replaced by a single-dose J&J vaccine which was shown to be more efficacious against severe COVID-19 in South Africa. The J&J vaccine was introduced by way of the SISONKE trial in health care workers, which was meant to establish real world effectiveness of the vaccine. In May 2021, Pfizer BioNTech was introduced in South Africa and made available alongside the J&J vaccine. In clinical trials Pfizer BioNTech showed efficacy of 95% against infection and severe disease, while J&J showed 72% efficacy (25, 26). The J&J vaccine has been shown to be 71% VEI and 94% effective against severe disease, hospitalization, and death (27). The Pfizer vaccine has been reported to be 88% VEI and 96% effective against severe disease, hospitalization, and death (28). Since we consider multiple vaccine candidates which have different reported and actual efficacy, we need to construct discrete distributions representing the different vaccine efficacy values. The discrete distributions for VEI, VES, and VEH for the J&J and Pfizer vaccines used in our computational study are available in Supplementary material.

#### 2.1.5 Relative susceptibility

In the model, we consider age-related differences in susceptibility to COVID-19. The *relative susceptibility* model parameter $\beta \left({\tilde{\omega }}_{c}\right)$ captures the variation in susceptibility due to the differences in social mixing and biological susceptibility between individuals in different age groups, i.e., A, B, C, and D. Current evidence suggests that there is age-dependent variation in susceptibility to COVID-19; susceptibility is elevated for adults over 65 years old and generally lower in the younger population. As in other parts of the world, in South Africa older adults, especially those aged 65 and older, were observed to be at a higher risk of severe illness, hospitalization, and death from COVID-19. Children and young adults were generally found to be less susceptible to severe outcomes compared to older age groups. In addition, underlying health conditions or comorbidities, such as cardiovascular disease, diabetes, and respiratory conditions, increased the risk of severe outcomes across all age groups (29, 30). The relative susceptibility discrete distributions used in our computational study are included in Supplementary material.

#### 2.1.6 Relative infectivity

The *relative infectivity* model parameter $\lambda \left({\tilde{\omega }}_{c}\right)$ captures the variation in infectiousness between infected individuals due to the differences in social mixing and biological infectivity between individuals in different age groups. Multiple studies suggest that there is little evidence that relative infectivity of older age groups is slightly higher than younger population (31). Infectivity is influenced by demographics, healthcare capacities and human mobility (32). Due to a lack of extensive studies on age-related differences in infectivity at the time of this study for COVID-19, we assume that the younger population has generally more human interactions that outweigh the older population's biological infectivity (33). Thus, the younger population has higher infectivity than the older population and does not develop severe symptoms as compared to older populations. For each municipality, we generated discrete distributions for relative infectivity which we used in our computational study shown in Supplementary material.

#### 2.1.7 Reliability level

**Figure 2:**
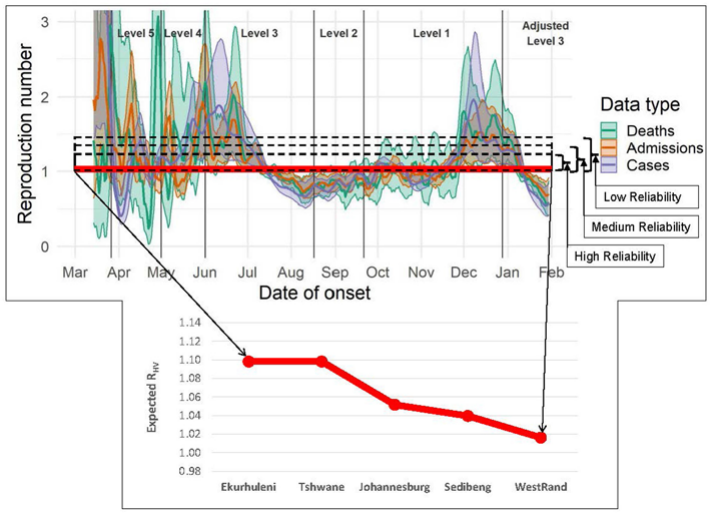
Estimated daily reproduction number *R*_*t*_, with 95% confidence intervals, Gauteng Province (last date included in the estimation: 30 Jan 2021) taken from (24) and overlaid with, **top**: Low, Medium, and High reliability levels α (or probabilistic risk tolerance) used in the integrated chance constraints stochastic programming (ICC-SP) model shown as dashed rectangles above *R*_*t*_ = 1; the horizontal red (thick) line is a plot of the ICC-SP model's optimal expected post-vaccination reproduction number *R*_*HV*_; and **bottom**: a closeup view of the plot of *R*_*HV*_ for each municipality in Gauteng Province showing that ICC-SP model's vaccination strategies are able to keep *R*_*HV*_ very close to one and thus, prevent epidemics.

The reliability level model parameter α is set by the public health authorities based on the historical severity of the epidemic. For this study, we experimented with three levels for reliability, namely *Low, Medium*, and *High*. The reason for doing this was to assess the sensitivity of the model to the historical range of values of the effective reproduction number. Based on the model data, we computed the excess *z*(ω) of the effective reproduction number above one for each municipality under each scenario ω with no vaccines at the lowest intervention Level 1. The expected excess $𝔼\left[z\left(\tilde{\omega }\right)\right]$ values for each municipality was then calculated. The acceptable expected excess α for each reliability level for a given community was then set as a fraction of $𝔼\left[z\left(\tilde{\omega }\right)\right]$. The reliability level can be thought of as a probabilistic risk tolerance, i.e., a quantifiable level of uncertainty or potential for a disease outbreak that the decision maker is willing to accept. Figure 2 top shows a plot of the Low, Medium, and High reliability levels, shown as dashed rectangles. The represent the maximum allowable tolerance above one of the reproduction number *R*_*HV*_ resulting from the vaccination strategy.

### 2.2 Multi-community stochastic model of disease spread

We begin our mathematical model description with some mathematical preliminaries in the next subsection necessary for the derivation of the model which follows thereafter.

#### 2.2.1 Preliminaries

ICC-SP was introduced by (34) and reduced forms of the model and an algorithm to solve the problem were derived in (35). ICC is a quantitative risk approach based on constraint violation as opposed to chance constraints SP (36–38), which is a qualitative risk approach. Specifically, ICC-SP was derived for production planning to meet uncertain demand in which the amount of constraint violation was quantified as a *shortage* (34). In our context of disease spread, we use ICC-SP to quantify the amount of constraint violation as an *excess* (surplus). Specifically, we are interested in bounding the excess of the expected reproduction number above one to prevent epidemics.

Let us begin with a generic ICC-SP formulation to provide a basis for the derivation of the multi-community disease spread model in the next section. Let $\tilde{\omega }$ denote a discretely distributed multivariate random variable whose outcomes (scenarios) are denoted by ω∈Ω, where the set Ω is the sample space. Each scenario ω has a probability of occurrence *p*(ω) and the set Ω is assumed to be finite. We can now define a generic SP as follows:


$$\begin{array}{cc} \text{Min} & \;g\left(x\right) \end{array}$$ (1a)

$$\begin{array}{cc} \text{s.t. } & Ax\geq b \end{array}$$ (1b)

$$\begin{array}{c} t{\left(\tilde{\omega }\right)}^{⊤}x\leq h\left(\tilde{\omega }\right) \end{array}$$ (1c)

$$\begin{array}{c} x\geq 0, \end{array}$$ (1d)

where $x\in {\mathbb{R}}_{+}^{n_{1}}$ is the decision variable vector, *g*(*x*) is the objective function, $A\in {\mathbb{R}}^{m_{1}}\times {\mathbb{R}}^{n_{1}}$ is a constraint matrix, and $b\in {\mathbb{R}}^{m_{1}}$ a right hand side vector. The left hand side coefficient vector $t\left(\tilde{\omega }\right)\in {\mathbb{R}}^{n_{1}}$ and right hand side scalar $h\left(\tilde{\omega }\right)\in \mathbb{R}$ are both random and thus, depend on $\tilde{\omega }$. The objective function (Equation 1a) is to minimize a linear cost function of the decisions *x* subject to Constraints (Equations 1b–1d). Constraint (Equation 1b) are deterministic constraints while Constraint (Equation 1c) is a random constraint. Constraint (Equation 1b) imposes non-negativity restrictions on *x*. Observe that since Constraint (Equation 1c) is random, there is a possibility of violating it. So letting


$$\eta \left(x,\tilde{\omega }\right):=t{\left(\tilde{\omega }\right)}^{⊤}x-h\left(\tilde{\omega }\right),$$

we define


$$\eta {\left(x,\tilde{\omega }\right)}^{+}:=\max\left\{0,\eta \left(x,\tilde{\omega }\right)\right\}.$$

We refer to the random amount $\eta {\left(x,\tilde{\omega }\right)}^{+}$ as the *excess* or surplus of violating the random constraint. Even though enforcing Constraint (Equation 1c) prevents having any excess, this may not be possible for some scenarios.

The fundamental idea of ICC is to allow for constraint violation up to an acceptable reliability level α, which is set by the modeler or decision-maker. To accomplish this, we can use the *mean excess* $𝔼\left[\eta {\left(x,\tilde{\omega }\right)}^{+}\right]$, where expectation *E* is taken with respect to $\tilde{\omega }$ over the sample space Ω. So instead of having Constraint (Equation 1c), we can now impose the following constraint instead:


$$\begin{array}{c} 𝔼\left[\eta {\left(x,\tilde{\omega }\right)}^{+}\right]\leq \alpha ,\;\alpha \in \left[0,\infty \right]. \end{array}$$ (2)

Constraint (Equation 2) is referred to as the ICC constraint. In the case when $\tilde{\omega }$ is a continuously distributed multivariate random variable, the expectation involves *integration* and thus, the name ICC. To obtain an ICC-SP model, we need to extend Model (Equation 1) to include Constraint (Equation 2). To that end, let *z*_ω_, ω∈Ω, represent the excess of violating Constraint (Equation 1c). Then, an ICC-SP model with finite discrete distribution can be written as follows:


$$\begin{array}{cc} \text{Min} & \;g\left(x\right)\\ \text{s.t. } & Ax\geq b \end{array}$$

$$\begin{array}{cc} \; & t{\left(\omega \right)}^{⊤}x-z\left(\omega \right)\leq h\left(\omega \right),\;\omega \in \Omega \end{array}$$ (3a)

$$\begin{array}{cc} \; & \underset{\omega \in \Omega }{\sum }p\left(\omega \right)z\left(\omega \right)\leq \alpha \end{array}$$ (3b)

$$\begin{array}{cc} \; & x,\;z\left(\omega \right)\geq 0,\;\omega \in \Omega . \end{array}$$ (3c)

Constraint (Equation 3a) requires *t*(ω)^⊤^*x* ≤ *h*(ω) for each ω∈Ω and if that is not possible, i.e., *t*(ω)^⊤^*x*>*h*(ω) for some ω, the model computes the excess *z*(ω) and bounds the mean excess by α in Constraint (Equation 3b). Constraint (Equation 3c) imposes non-negativity restrictions on the decision variables. We should point out that Model (Equation 3) is a large-scale linear program (LP) and may be solved using state-of-art optimization solvers. However, when the number of scenarios |Ω| is very large the model can be computationally challenging to solve without using decomposition methods.

The idea of ICC naturally extends to the optimal vaccine allocation setting to prevent epidemics. A vaccination campaign and/or intervention measures such as lockdowns and social distancing may not prevent an epidemic. In epidemiology, the relation between vaccination and the reproduction number *R*_0_ is well understood (11, 39, e.g.,). As pointed out in the previous section, a value of *R*_0_ greater than one means that the disease will continue to spread, i.e., there is an epidemic. During an epidemic (or pandemic) *R*_0_ is estimated using the *effective* reproduction number *R*_*t*_, which is uncertain at best. Thus, it is a random variable and we can write it as a function of $\tilde{\omega }$, i.e., $R_{t}\left(\tilde{\omega }\right)$. In the case of a vaccination campaign, we are interested in *R*_0_ after vaccination, i.e., the *post-vaccination* reproduction number of infected households *R*_*HV*_. This is also a random variable and we can write it as $R_{HV}\left(\tilde{\omega }\right)$. The goal of a vaccination campaign is to attain a vaccination coverage that induces herd immunity in the population so that $R_{HV}\left(\tilde{\omega }\right)\leq 1$. However, achieving $R_{HV}\left(\tilde{\omega }\right)\leq 1$ may not be possible under all scenarios. Therefore, we can naturally apply ICC to determine an optimal vaccination coverage that provides the mean excess of $R_{HV}\left(\tilde{\omega }\right)$ above one below a given reliability level α∈[0, *r*], where *r* <∞. The random *excess* is given as


$$\eta {\left(x,\tilde{\omega }\right)}^{+}:=\max\left\{0,R_{HV}\left(\tilde{\omega }\right)-1\right\}.$$

Then using Constraint (Equation 2), we can write the ICC constraint for bounding the mean excess of the post-vaccination reproduction number above one as follows:


$$\begin{array}{c} 𝔼\left[\eta {\left(x,\tilde{\omega }\right)}^{+}\right]\leq \alpha ,\;\alpha \in \left[0,r\right]. \end{array}$$ (4)

In reality, the effective reproduction number *R*_*t*_ is finite and so is the excess $\eta {\left(x,\tilde{\omega }\right)}^{+}$. Thus, we want to keep *r* as small as possible to ensure that $R_{HV}\left(\tilde{\omega }\right)$ is below one for most of the scenarios that are likely to occur.

We can now extend the generic ICC-SP Model (Equation 3) to the vaccination setting as follows:


$$\begin{array}{cc} \text{Min } & g\left(x\right) \end{array}$$ (5a)

$$\begin{array}{cc} \text{s.t. } & Ax\geq b \end{array}$$ (5b)

$$\begin{array}{cc} \; & R_{HV}{\left(\omega \right)}^{⊤}x-z\left(\omega \right)\leq 1,\;\omega \in \Omega \end{array}$$ (5c)

$$\begin{array}{cc} \; & \underset{\omega \in \Omega }{\sum }p\left(\omega \right)z\left(\omega \right)\leq \alpha \end{array}$$ (5d)

$$\begin{array}{cc} \; & x,\;z\left(\omega \right)\geq 0,\;\omega \in \Omega . \end{array}$$ (5e)

The decision *x* is the vaccination strategy, i.e., the proportion to vaccinate in each heterogeneous group. The objective function (Equation 5a) minimizes vaccine coverage, expressed using the cost function *g*(*x*). Constraint (Equation 5a) is the set of deterministic constraints such as budgetary constraints and/or constraints on the number of available vaccines. ICC constraint (Equation 4) is captured by Constraints (Equations 5c, 5d). Constraint (Equation 5c) calculates excess of the post-vaccination reproduction number above one for each scenario *z*(ω) while Constraint (Equation 5d) bounds the mean excess by the reliability level α.

#### 2.2.2 Model formulation

We use a stochastic extension of (11)'s deterministic epidemiology model for multi-community infectious disease spread to formulate our ICC-SP model to determine optimal vaccination strategies. The disease spread model can be *homogeneous* or *heterogeneous* depending on whether there are differences in the susceptibility to disease and/or infectivity of individuals based on biological characteristics. We consider the heterogeneous case and assume differences in disease susceptibility and infectivity for COVID-19 based on age. Thus, a community in our model involves households composed of heterogeneous individuals who are either vaccinated or unvaccinated. The model considers both *outside* and *within* household disease transmission by a susceptible individual in a household having a close contact with an infected individual outside the household. Within the household, the infected individual can transmit the disease to members of the household at a certain rate. The proposed ICC-SP model provides an advance over a previous model (16) in determining optimal vaccination strategies in two unique ways: (i) incorporates VEI, VES, and VEH; and (ii) includes intervention levels (*Level 1, Level 2, Level 3, Level 4*, and *Level 5*). Thus, the model determines not only the optimal vaccination strategy for each community, but also the optimal intervention level.

We are now in a position to derive the ICC-SP model. We use the mathematical notation defined in Table 3 to formulate the model. The fundamental idea of the ICC-SP model is to determine optimal vaccination strategies such that the post-vaccination reproduction number of infected households for each community *c*∈ℂ, $R_{HVc}\left(\tilde{\omega }\right)$, is bounded close to one. Since $R_{HVc}\left(\tilde{\omega }\right)$ is a random variable we need to bound the expected excess of $R_{HVc}\left(\tilde{\omega }\right)$ above one not to exceed the reliability level α_*c*_, i.e., we enforce the requirement


$$𝔼\left[\eta {\left(x,\tilde{\omega }\right)}^{+}\right]\leq \alpha_{c},$$

**Table 3:** Notation used for the stochastic model of disease spread.

**Sets and indices**	
ℂ	Index set of communities, element *c*∈ℂ.
ℕ	Index set of household types, element *n*∈ℕ.
*I*	Index set of person age groups, element *i*∈*I*.
*L*	Index set of intervention levels, *L* = {1, ⋯ , 5}, element ℓ∈*L*.
Ω_*c*_	Index set of scenarios for community *c*∈ℂ, element ω_*c*_∈Ω_*c*_.
**Parameters**	
${\tilde{\omega }}_{c}$	Multivariate random variable whose outcome ω_*c*_∈Ω_*c*_ describes the uncertain disease spread parameters for the reproduction number.
$R_{HVc}\left({\tilde{\omega }}_{c}\right)$	Post-vaccination reproduction number of infected households for community *c*∈ℂ under scenario ${\tilde{\omega }}_{c}$.
$a_{nj\ell c}\left({\tilde{\omega }}_{c}\right)$	Uncertain parameter that captures the impact of vaccination strategy
	*j* in a type *n* household at intervention level ℓ∈*L* in community *c*∈ℂ.
$m_{\ell c}\left({\tilde{\omega }}_{c}\right)$	Uncertain number of close contacts that an infective makes on average with persons from other household in the course of his/her infectious period in a community *c*∈ℂ under intervention level ℓ∈*L*.
*p*(*n*)	Number of persons in a household of type *n*∈ℕ.
*p*_*i*_(*n*)	Number of persons in age group *i*∈{*A, B, C, D*} in a household of type *n*∈ℕ.
*f*_*i*_(*n, j*)	Number of persons to vaccinate in age group *i*∈{*A, B, C, D*} in household of type *n*∈ℕ when vaccination strategy *j*∈{1, ⋯ , *J*(*n*)} is implemented, *f*_*i*_(*n, j*) ≤ *p*_*i*_(*n*).
*v*(*n, j*)	Vaccination strategy for type *n* household, *v*(*n, j*) = (*f*_*A*_(*n, j*), *f*_*B*_(*n, j*), *f*_*C*_(*n, j*), *f*_*D*_(*n, j*)), which is the number of persons to vaccinate in each age group, *j*∈{1, ⋯ , *J*(*n*)}.
*H*_*c*_	Number of households in community *c*∈ℂ.
*h*_*nc*_	Proportion of type *n* households in community *c*∈ℂ.
μ_*c*_	Average household size in a community, $\mu_{c}=\underset{n\in \mathbb{N}}{\sum }p\left(n\right)h_{nc}$.
$b\left({\tilde{\omega }}_{c}\right)$	Uncertain transmission rate within a household.
$\beta_{ic}\left({\tilde{\omega }}_{c}\right)$	Uncertain susceptibility for age group *i*∈*I* in community *c*∈ℂ.
$\lambda_{ic}\left({\tilde{\omega }}_{c}\right)$	Uncertain infectivity for age group *i*∈*I* in community *c*∈ℂ.
$\epsilon \left({\tilde{\omega }}_{c}\right)$	Uncertain vaccine efficacy toward population in community *c*∈ℂ.
*M*	A large number at least equal to the maximum value of the effective reproduction number.
*M*_ℓ*c*_	Penalty factor for intervention level ℓ∈*L* for community *c*∈ℂ.
α_*c*_	User-set model reliability level for community *c*∈ℂ.
γ	User-set model weight factor for intervention level preference.
*V*	Total number of available vaccines.
*x*_*njc*_	Proportion of *n* sized households under vaccination strategy *j*∈{1, ⋯ , *J*(*n*)} implemented
	in community *c*∈ℂ.
*y*_ℓ*c*_	*y*_ℓ*c*_ = 1 if intervention level for community *c*∈ℂ is at ℓ∈*L*, *y*_ℓ*c*_ = 0 otherwise.
*z*_ω*ℓc*_	Excess/surplus of reproduction number above one for scenario ω∈Ω at intervention
	level ℓ∈*L* for community *c*∈ℂ.

where $\eta {\left(x,\tilde{\omega }\right)}^{+}:=\max\left\{0,R_{HVc}\left(\tilde{\omega }\right)-1\right\}$. Furthermore, we seek vaccination strategies that provide the minimum vaccine coverage *g*(*x*) at the lowest intervention level ℓ∈*L* possible. The parameter α_*c*_ is determined and set by the public health authorities based on how the disease is spreading. Clearly, $R_{HVc}\left(\tilde{\omega }\right)$ is unknown and computing it requires the disease spread parameter $a_{nj\ell c}\left({\tilde{\omega }}_{c}\right)$, which is uncertain and captures the impact of a vaccination strategy *j* in a type *n*∈ℕ household at intervention level ℓ in community *c*∈ℂ. We shall explain what we mean by a *vaccination strategy* momentarily. The explicit expression for $R_{HVc}\left({\tilde{\omega }}_{c}\right)$ is derived from Becker and Starczak's deterministic model (11), where all the model parameters are assumed to be known. In the ICC-SP model, however, the parameters are assumed to be unknown and are described by a multivariate random variable ${\tilde{\omega }}_{c}$ whose probability distribution can be estimated using available disease spread data. Thus, the model parameters depend on the outcome (scenario) ω_*c*_ of ${\tilde{\omega }}_{c}$. A *scenario* ω_*c*_ is described by the quintuple: ω_*c*_: = {*m*_ℓ*c*_(ω_*c*_), *b*(ω_*c*_), ϵ(ω_*c*_), β_*ic*_(ω_*c*_), λ_*ic*_(ω_*c*_)} and probability of occurrence *p*(ω_*c*_). The elements of the quintuple specify the outcomes of *average contact rate outside the household, within household contact rate, vaccine efficacy, relative susceptibility*, and *relative infectivity*. Vaccine efficacy can be specified as VEI, VES, or VEH. This provides versatility for public health authorities in terms of choosing which vaccine efficacy criterion to use in determining optimal vaccination strategies.

Following (11), the parameter *a*_*njℓc*_(ω_*c*_) for scenario ω_*c*_ is expressed as


$$\begin{array}{l} a_{njlc}(\omega_{c})=\frac{m_{lc}(\omega_{c})h_{nc}}{\mu_{c}}(\sum_{i\in I}\beta_{ic}(\omega_{c})\lambda_{ic}(\omega_{c})[(1-b(\omega_{c}))(p_{i}(n)\\ \;-f_{i}(n,j)\epsilon (\omega s_{c}))\;+b(\omega_{c})f_{i}(n,j)\epsilon (\omega_{c})(1-\\ \;\epsilon (\omega_{c}))]+b({\tilde{\omega }}_{c})\sum_{i\in I}\sum_{r\in I}\beta_{ic}(\omega_{c})\lambda_{rc}(\omega_{c})[p_{i}(n)\\ \;-f_{i}(n,j)\epsilon (\omega_{c})](p_{r}(n)-f_{r}(n,j)\epsilon (\omega_{c}))). \end{array}$$ (6)

Equation 6 can be explained as follows: The term outside the outer parenthesis captures disease spread outside the household, which involves the product of the outside close contact rate *m*_ℓ*c*_(ω_*c*_) and the proportion of type *n* households in the community *h*_*nc*_ divided by the average household size in the community μ_*c*_. Notice that outside close contact rate depends on the scenario ω_*c*_ and the prevailing intervention level ℓ∈*L*. The terms inside the outer parenthesis capture disease spread within the household based on how the disease spreads between the members based on their ages and whether they are vaccinated or not. This involves the product terms that include relative susceptibility β_*ic*_(ω_*c*_), infectivity λ_*ic*_(ω_*c*_), within household contact rate *b*(ω_*c*_), and vaccine efficacy ϵ(ω_*c*_). Disease spread within the household is driven by members who get infected outside the household and then have close contacts within the household. As the computational study results show later, having relatively high outside close contact rate coupled with high relative susceptibility β_*ic*_(ω_*c*_) and infectivity favors faster disease spread in the community.

To formulate the ICC-SP model, let *x, y*, and *z* denote decision variable vectors that are appropriately dimensioned with components *x*_*njc*_, *y*_ℓ*c*_, and *z*_ω*ℓc*_, respectively. The decision variable *x* specifies the proportion of *n* sized households under vaccination strategy *j*∈{1, ⋯ , *J*(*n*)} for community *c* while *y* specifies the intervention level, with *y*_ℓ*c*_ = 1 if intervention level for community *c* is at level ℓ, and *y*_ℓ*c*_ = 0 otherwise. The decision variable *z* specifies the excess of the post-vaccination reproduction number above one for scenario ω at intervention level ℓ for community *c*. Before we state and describe the ICC-SP formulation, let us first define a *vaccination strategy*. Simply put, a vaccination strategy is a specification of the number of persons to vaccinate in each age group. Let *n* = 1, 2, 3, ⋯  denote the household type and *p*(*n*) be the corresponding household size. For each household type *n* we have the household composition (*p*_*A*_(*n*), *p*_*B*_(*n*), *p*_*C*_(*n*), *p*_*D*_(*n*)), where *p*_*i*_(*n*) is the number of household members that belong to age group *i*∈{*A, B, C, D*}. Thus, the total number of vaccination policies for a household type *n*, *J*(*n*), is given by


$$J\left(n\right)=\left(p_{A}\left(n\right)+1\right)*\left(p_{B}\left(n\right)+1\right)*\left(p_{C}\left(n\right)+1\right)*\left(p_{D}\left(n\right)+1\right).$$

A *vaccination strategy* for a type *n* household is denoted by *v*(*n, j*), *j* = 1, ⋯ , *J*(*n*) and specifies the number of persons to vaccinate in each age group, *f*_*i*_(*n, j*), *i*∈{*A, B, C, D*}. It is given as follows:


$$v\left(n,j\right)=\left(f_{A}\left(n,j\right),f_{B}\left(n,j\right),f_{C}\left(n,j\right),f_{D}\left(n,j\right)\right).$$

Notice that for each *i*∈{*A, B, C, D*}, *f*_*i*_(*n, j*) ≤ *p*_*i*_(*n*). We illustrate the concept of a vaccination strategy in Table 4 for household sizes one and two. As shown in the table, household sizes one and two results in vaccination policies for household types *n* = 1, ⋯ , 14.

**Table 4:** Example household types and vaccination policies under heterogeneous population for household size one and size two.

**Household type *n***	**Household size *p*(*n*)**	**Household composition**	**Total vaccination policies *J*(*n*)**	**Possible vaccination policies for a type *n* Household *v*(*n, j*)**
		(*p*_*A*_(*n*),	(*p*_*A*_(*n*)+1)*	(*f*_*A*_(*n, j*), *f*_*B*_(*n, j*), *f*_*C*_(*n, j*), *f*_*D*_(*n, j*))
		*p*_*B*_(*n*),	(*p*_*B*_(*n*)+1)*	
		*p*_*C*_(*n*),	(*p*_*C*_(*n*)+1)*	
		*p*_*D*_(*n*))	(*p*_*D*_(*n*)+1)	
1	1	(1, 0, 0, 0)	2	(0, 0, 0, 0), (1, 0, 0, 0)
2	1	(0, 1, 0, 0)	2	(0, 0, 0, 0), (0, 1, 0, 0)
3	1	(0, 0, 1, 0)	2	(0, 0, 0, 0), (0, 0, 1, 0)
4	1	(0, 0, 0, 1)	2	(0, 0, 0, 0), (0, 0, 0, 1)
5	2	(2, 0, 0, 0)	3	(0, 0, 0, 0), (1, 0, 0, 0), (2, 0, 0, 0)
6	2	(0, 2, 0, 0)	3	(0, 0, 0, 0), (0, 1, 0, 0), (0, 2, 0, 0)
7	2	(0, 0, 2, 0)	3	(0, 0, 0, 0), (0, 0, 1, 0), (0, 0, 2, 0)
8	2	(0, 0, 0, 2)	3	(0, 0, 0, 0), (0, 0, 0, 1), (0, 0, 0, 2)
9	2	(1, 1, 0, 0)	4	(0, 0, 0, 0), (0, 1, 0, 0), (1, 0, 0, 0), (1, 1, 0, 0)
10	2	(0, 1, 1, 0)	4	(0, 0, 0, 0), (0, 0, 1, 0), (0, 1, 0, 0), (0, 1, 1, 0)
11	2	(0, 0, 1, 1)	4	(0, 0, 0, 0), (0, 0, 1, 0), (0, 0, 0, 1), (0, 0, 1, 1)
12	2	(1, 0, 1, 0)	4	(0, 0, 0, 0), (1, 0, 0, 0), (0, 0, 1, 0), (1, 0, 1, 0)
13	2	(1, 0, 0, 1)	4	(0, 0, 0, 0), (1, 0, 0, 0), (0, 0, 0, 1), (1, 0, 0, 1)
14	2	(0, 1, 0, 1)	4	(0, 0, 0, 0), (0, 1, 0, 0), (0, 0, 0, 1), (0, 1, 0, 1)

Now, turning to the formulation of the ICC-SP model, the notion of a vaccination strategy is central to how the model will determine the values the decision variables *x, y*, and *z* should take. Observe that we have a combinatorial problem in the sense that the model has to enumerate all vaccination policies for the given problem data to determine the optimal proportion of type *n* households to assign to each vaccination strategy for each community (decision *x*). At the same time, the model must determine the optimal intervention level (decision *y*) at which the excess of the reproduction number above one is below or equal to the specified reliability level α_*c*_ (decision *z*). Given the parameter *a*_*njℓc*_(ω) (calculated based on Equation 6) for all scenarios ω∈Ω, we can write the ICC-SP model with *unlimited vaccines* as follows:


$$\begin{array}{cc} \underset{x,y,z}{\text{Min}}\; & g\left(x,y\right):=\underset{c\in \mathbb{C}}{\sum }\underset{n\in \mathbb{N}}{\sum }\overset{J\left(n\right)}{\underset{j=0}{\sum }}\underset{i\in I}{\sum }f_{i}\left(n,j\right)h_{nc}x_{njc}+\gamma \underset{c\in \mathbb{C}}{\sum }\underset{\ell \in L}{\sum }M_{\ell c}y_{\ell c} \end{array}$$ (7a)

$$\begin{array}{cc} \text{s.t. } & \underset{n\in \mathbb{N}}{\sum }\overset{J\left(n\right)}{\underset{j=0}{\sum }}a_{nj\ell c}\left(\omega \right)x_{njc}-z_{\omega \ell c}\leq 1,\;\forall \;\omega \in \Omega ;\;\ell \in L;c\in \mathbb{C} \end{array}$$ (7b)

$$\begin{array}{c} \underset{\omega \in \Omega }{\sum }p_{\omega }z_{\omega \ell c}+My_{\ell c}-\alpha_{c}y_{\ell c}\leq M,\;\forall \;\ell \in L;c\in \mathbb{C} \end{array}$$ (7c)

$$\begin{array}{c} \overset{J\left(n\right)}{\underset{j=0}{\sum }}x_{njc}=1,\;\forall \;n\in \mathbb{N};\;c\in \mathbb{C} \end{array}$$ (7d)

$$\begin{array}{c} \underset{\ell \in L}{\sum }y_{\ell c}=1,\;\forall \;c\in \mathbb{C} \end{array}$$ (7e)

$$\begin{array}{l} x_{njc},z_{\omega \ell c}\geq 0,y_{\ell c}\in \left\{0,1\right\},\forall \;n\in \mathbb{N};j\in \left\{0,\cdots ,J\left(n\right)\right\};\\ \omega \in \Omega ;\;\ell \in L;\;c\in \mathbb{C}. \end{array}$$ (7f)

The Objective Function (Equation 7a) minimizes the total vaccination coverage plus a weighted penalty cost for the intervention level. The goal of the model is to prevent epidemics at the lowest level of intervention possible. Constraint (Equation 7b) computes the post-vaccination reproduction number and forces it to be below one if possible. If it is not below one, the constraint computes the excess above one for the scenario and intervention level. The expected excess for each intervention level for a given community is calculated in Constraint (Equation 7c). Constraint (Equation 7d) enforces the requirement that the proportions of the *n*-sized households vaccinated under each vaccination strategy sum to one for each community, while Constraint (Equation 7e) ensures that one intervention level is selected for each community. Finally, Constraint (Equation 7f) enforces non-negativity restrictions on the decision variables *x* and *z*, and binary restrictions on the *y* decision variables.

In practice, there will be a limited number of available vaccines. Therefore, the ICC-SP Problem 7 can be extended to include this restriction by adding the following constraint:


$$\begin{array}{c} \underset{c\in \mathbb{C}}{\sum }H_{c}\underset{n\in \mathbb{N}}{\sum }h_{nc}\overset{J\left(n\right)}{\underset{j=0}{\sum }}\underset{i\in I}{\sum }\frac{f_{i}\left(n,j\right)}{p\left(n\right)}x_{njc}\leq V, \end{array}$$ (8)

where *V* is the total number of vaccines available. Constraint (Equation 8) computes the total number of vaccines needed to prevent epidemics in each community if possible, and enforces this number to be below *V*, which is the total number of vaccines available. This constraint also enables computing the number of vaccines allocated to each community. Notice that Constraint (Equation 8) links all the communities and the formulation is no longer separable by communities. Consequently, solving the limited vaccines problem can be more challenging than solving Problem (Equation 7).

## 3 Results

We implemented the ICC-SP model to investigate the effectiveness of COVID-19 vaccination strategies under vaccination effectiveness against infection (VEI), hospitalization (VEH), and symptomatic (VES) cases using data for Gauteng province described in Section 2.1. The population data for the municipalities used in this study is as follows: Ekurhuleni (4,024,285), Johannesburg (5,079,469), Sedibeng (1,125,281), Tshwane (3,832,516), and West Rand (1,007,756) (20). The ICC-SP model was coded using C++ in Microsoft Visual Basic in the IBM ILOG CPLEX 12.9 Callable Library environment (40). We chose this software environment to enable implementation of a cutting plane decomposition method based on (35) to solve instances of ICC-SP. We created several instances of the model based on a set of predetermined reliability levels α_*c*_ to generate optimal vaccination strategies and intervention levels for the five municipalities. All the experiments were conducted on a computer workstation with 4.2 GHz CPU with 16 Cores and 64 GB of RAM. Due to the large-scale nature of the ICC-SP because of having multiple communities, household types, scenarios, and binary decision variables, we used the cutting-plane decomposition method to solve the instances. For the provincial level instances that we created, the average computation (CPU) time to run each ICC-SP problem instance is around 300 seconds with a wall clock time of about one hour. Thus, the ICC-SP model is suitable for up to the provincial level real-time (hourly) optimal decision making and can be used for real-time or near-real-time policy (minutes) requiring good feasible solutions. Scalability to larger regions or national level models would require scenario reduction techniques and other approximations, and further improvements in the decomposition solution approach.

We experimented with the three levels for reliability defined in Section 2.1, namely *Low, Medium*, and *High* as illustrated in Figure 1. The reliability levels allowed for assessing the ICC-SP model's optimal solutions across the historical range of values of *R*_*t*_ (effective reproduction number) above one (i.e., *z*_ω*ℓc*_) for each municipality under no vaccines at the lowest intervention ℓ (Level 1). The range of values of *z*_ω*ℓc*_ for each municipality across all scenarios after solving the ICC-SP model are as follows: Ekurhuleni [0, 4.16], Johannesburg [0, 4.00], Sedibeng [0, 4.14], Tshwane [0, 4.12], and West Rand [0, 4.16]. The acceptable expected excess α_*c*_ value for each municipality *c* and reliability level was calculated via experimentation as a percentage of $𝔼\left[z\left(\tilde{\omega }\right)\right]$. *High* reliability was assigned the smallest value, followed by *Medium* reliability, and then *Low* reliability as shown in the table. We experimented with limited vaccine availability of up to two-thirds of the total population of the five municipalities. To consider age-related differences in infectivity of COVID-19, we investigated two cases in our experiments. In the first case, we experimented with age-related differences in infectivity such that Group *D* has lower relative infectivity than Groups *C*, *B*, and *A*, in that order (younger population is more infective). In the second case, we considered Group *A* with lower relative infectivity than Groups *B*, *C*, and *D*, in that order (older population is more infective). We present detailed results for the first case and then discuss the key findings for both cases.

We first ran the ICC-SP model without fixing intervention levels to determine both the optimal proportion of the population to vaccinate to prevent epidemics and the optimal intervention level ℓ for each community and reliability level α_*c*_. The results are summarized in Table 5. Under VEI as the vaccine efficacy criterion and Low reliability (i.e., relatively high risk in terms of preventing epidemics) the optimal intervention level to prevent epidemics across all municipalities is Level 1 (lowest). However, the optimal proportion of the population to vaccinate is largest for Ekurhuleni and Tshwane (round 61%), followed by Johannesburg and Sedibeng (around 58%), and then West Rand (around 45%). Under Medium reliability (i.e., relatively medium risk in terms of preventing epidemics) the optimal intervention levels to prevent epidemics from highest to lowest are as follows: Tshwane at Level 3 (round 63%), Ekurhuleni at Level 3 (round 62%), Johannesburg at Level 2 (around 72%), Sedibeng at Level 1 (around 78%), and West Rand at Level 1 (around 58%). Under High reliability (i.e., relatively low risk in terms of preventing epidemics) the optimal intervention levels to prevent epidemics from highest to lowest are as follows: Ekurhuleni and Tshwane both at Level 4 (round 54%), Johannesburg at Level 3 (around 76%), Sedibeng at Level 2 (around 93%), and West Rand at Level 1 (around 71%). In terms of general trends across reliability levels, we can see that both Ekurhuleni and Tshwane require the largest proportion to vaccine as well as high intervention levels under both Medium and High reliability levels, followed by Johannesburg, Sedibeng, and then West Rand. We also observe that the proportion to vaccinate under both Medium and High reliability exceed 70% of the population for some of the municipalities. Such a high proportion to vaccinate may not be attainable in practice and would be an indication that the epidemics may not be prevented.

**Table 5:** Optimal proportion (%) of population to vaccinate along with optimal intervention level for each municipality under VEI, VES, and VEH as the vaccine efficacy criteria.

**Municipality *c***	***%* Vac**.	**Level ℓ**	***%* Vac**.	**Level ℓ**	***%* Vac**.	**Level ℓ**
	**VEI Reliability Level**					
	**Low**, $\alpha_{c}=25\%\times 𝔼\left[z\left(\tilde{\omega }\right)\right]$		**Medium**, $\alpha_{c}=17.5\%\times 𝔼\left[z\left(\tilde{\omega }\right)\right]$		**High**, $\alpha_{c}=12.5\%\times 𝔼\left[z\left(\tilde{\omega }\right)\right]$	
Ekurhuleni	60.6	1	61.9	3	53.5	4
Johannesburg	58.1	1	72.4	2	75.7	3
Sedibeng	58.0	1	77.7	1	93.4	2
Tshwane	61.6	1	62.7	3	54.3	4
West Rand	44.6	1	57.5	1	71.4	1
	**VES Reliability Level**					
	**Low**, $\alpha_{c}=7.5\%\times 𝔼\left[z\left(\tilde{\omega }\right)\right]$		**Medium**, $\alpha_{c}=5\%\times 𝔼\left[z\left(\tilde{\omega }\right)\right]$		**High**, $\alpha_{c}=2.5\%\times 𝔼\left[z\left(\tilde{\omega }\right)\right]$	
Ekurhuleni	67.6	1	69.9	2	72.7	3
Johannesburg	65.0	1	59.4	3	54.5	4
Sedibeng	64.4	1	70.8	1	81.1	1
Tshwane	67.8	1	74.6	1	73.0	3
West Rand	50.7	1	55.9	1	63.8	1
	**VEH Reliability Level**					
	**Low**, $\alpha_{c}=0.1\%\times 𝔼\left[z\left(\tilde{\omega }\right)\right]$		**Medium**, $\alpha_{c}=0.001\%\times 𝔼\left[z\left(\tilde{\omega }\right)\right]$		**High**, $\alpha_{c}=0.0\%\times 𝔼\left[z\left(\tilde{\omega }\right)\right]$	
Ekurhuleni	65.3	1	67.8	1	67.7	1
Johannesburg	64.3	1	66.3	1	66.3	1
Sedibeng	65.5	1	68.7	1	68.8	1
Tshwane	65.9	1	67.9	1	68.2	1
West Rand	52.9	1	56.6	1	56.7	1

% Vac, Proportion to vaccinate.

We performed a preliminary empirical analysis toward validating the ICC-SP model against observed outcomes by linking model outputs to the observed effective reproduction number *R*_*t*_ trends. We used the COVID-19 estimated daily *R*_*t*_ trends data for Gauteng province, South Africa, that are available at (24). The data starts from March of 2020 around the onset of the COVID-19 pandemic. In Figure 1, we plot the expected post-vaccination reproduction number *R*_*HV*_ obtained from the ICC-SP model against the observed daily *R*_*t*_ trends in Gauteng. As can be seen from the graph, the ICC-SP model is able to bring down the *R*_*t*_ values very close to one, an indication that the vaccination strategies from the model are able to reduce the spread of the disease as desired. We provide a detailed view of the ICC-SP model expected *R*_*HV*_ values for each of the five municipalities in Gauteng at the bottom of Figure 1. The minimum and maximum *R*_*HV*_ values obtained from the ICC-SP model for each municipality are as follows: Ekurhuleni (1.09, 1.12), Tshwane (1.09, 1.12), Johannesburg (1.04, 1.07), Sedibeng (1.02, 1.07), and West Rand (1.01, 1.03).

When VES is used as the vaccine efficacy criterion, we observe similar trends in terms of having elevated intervention levels and relatively large proportions to vaccinate under Medium and High reliability levels for Ekurhuleni, Johannesburg, and Tshwane. Sedibeng and West Rand both have the lowest intervention level and the proportion to vaccinate; albeit the proportion to vaccine for Sedibeng is around 70% under Medium reliability and just over 80% under High reliability. These percentages may not to be feasible in reality. Under Low reliability, however, all municipalities are at intervention Level 1 and require around 65% or more to vaccinate except for West Rand, which require around 50%. These proportions are more likely to be attainable in practice. Interestingly, when VEH is used as the vaccine efficacy criterion, all municipalities remain at intervention Level 1 and in this case, all the municipalities but one (West Rand) require vaccinating between 60% and 70% of the population under all the three reliability levels. West Rand requires vaccinating between 50% and 60% of its population.

**Figure 3:**
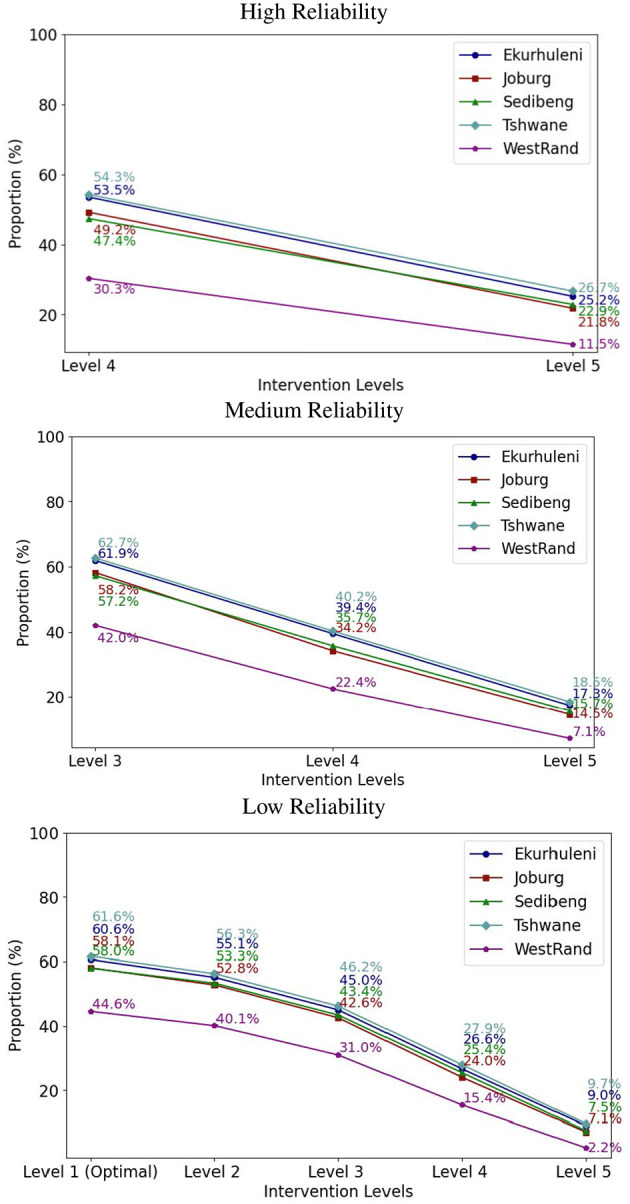
The integrated chance constraints stochastic programming (ICC-SP) model optimal proportion to vaccinate at each intervention level with vaccination against infection (VEI) as the vaccine efficacy criterion. The curves are for each municipality in Gauteng Province: Ekurhuleni (dark blue), Joburg (Johannesburg, maroon), Sedibeng (green), Tshwane (light blue), and West Rand (purple). The number above each curve is the percentage (%) of the population in the municipality to vaccinate to prevent epidemics.

**Figure 4:**
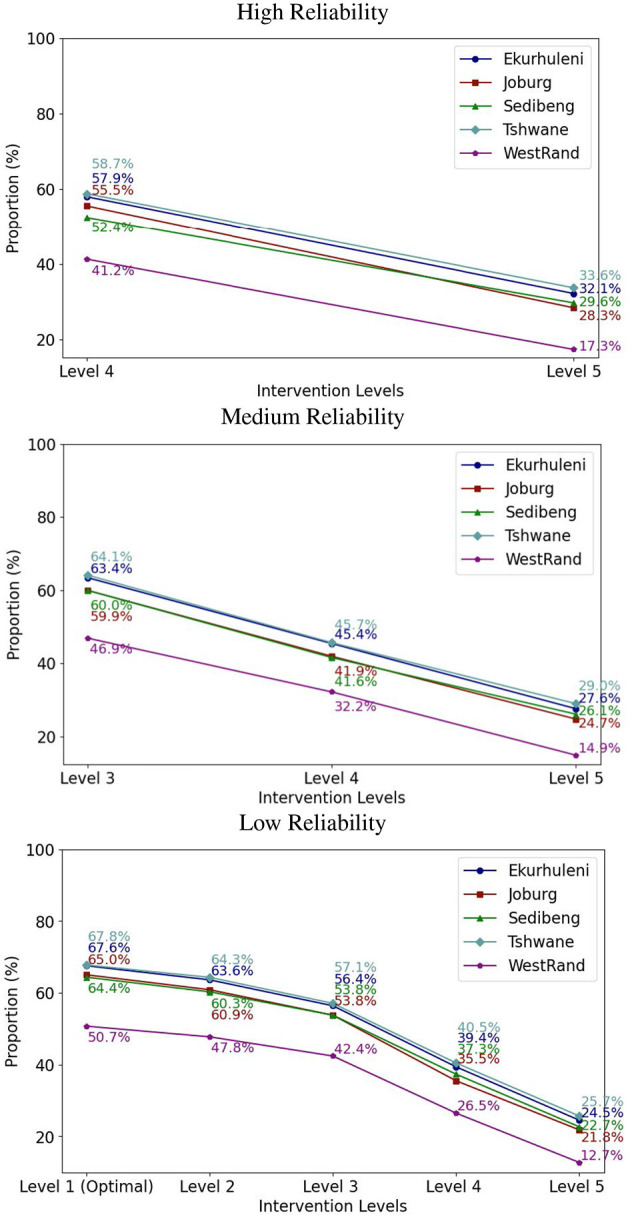
The integrated chance constraints stochastic programming (ICC-SP) model optimal proportion to vaccinate at each intervention level with vaccination against symptomatic cases (VES) as the vaccine efficacy criterion. The curves are for each municipality in Gauteng Province: Ekurhuleni (dark blue), Joburg (Johannesburg, maroon), Sedibeng (green), Tshwane (light blue), and West Rand (purple). The number above each curve is the percentage (%) of the population in the municipality to vaccinate to prevent epidemics.

**Figure 5:**
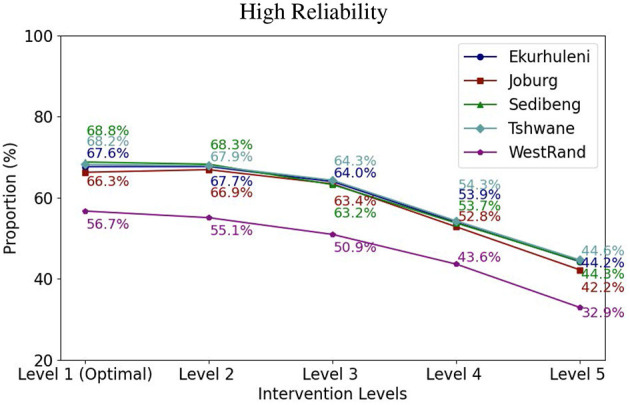
The integrated chance constraints stochastic programming (ICC-SP) model optimal proportion to vaccinate at each intervention level with vaccination against hospitalization (VEH) as the vaccine efficacy criterion. The curves are for each municipality in Gauteng Province: Ekurhuleni (dark blue), Joburg (Johannesburg, maroon), Sedibeng (green), Tshwane (light blue), and West Rand (purple). The number above each curve is the percentage (%) of the population in the municipality to vaccinate to prevent epidemics.

In practice, the same intervention level is usually applied across multiple communities to effectively contain the epidemics. Therefore, we experimented with fixing the intervention level in the ICC-SP model across the five municipalities to determine the optimal proportion to vaccinate to prevent epidemics at a given intervention level. The results for the three reliability levels for VEI, VES, and VEH are plotted in Figures 3–5, respectively. As can be seen in the plots in Figure 3 with VEI as the vaccine efficacy criterion, all five intervention levels are possible under Low reliability level. However, under Medium reliability only intervention Levels 3, 4, and 5 are possible. Under High reliability only Levels 4 and 5 are possible. We observe that, under Medium reliability, the epidemics cannot be controlled with intervention Levels 1 and 2. Similarly, under High reliability the epidemics cannot be prevented with intervention Levels 1, 2, and 3. Regarding the proportion of the population to vaccinate in each municipality to prevent epidemics, we observe from the plots that the proportion decreases with increasing intervention level as expected. We also notice that Ekurhuleni and Tshwane require the largest proportion to vaccinate under all three vaccine efficacy criteria (VEI, VES, and VEH) followed by Johannesburg and Sedibeng. West Rand requires the least proportion to vaccinate across all intervention levels. In terms of reliability levels from Low to High for a fixed intervention level, we can see that larger proportions have to be vaccinated to prevent epidemics. For example, Ekurhuleni under intervention Level 4 requires vaccinating 26.6% of the population to prevent epidemics under Low reliability, 39.4% under Medium reliability, and 53.5% High reliability.

**Figure 6:**
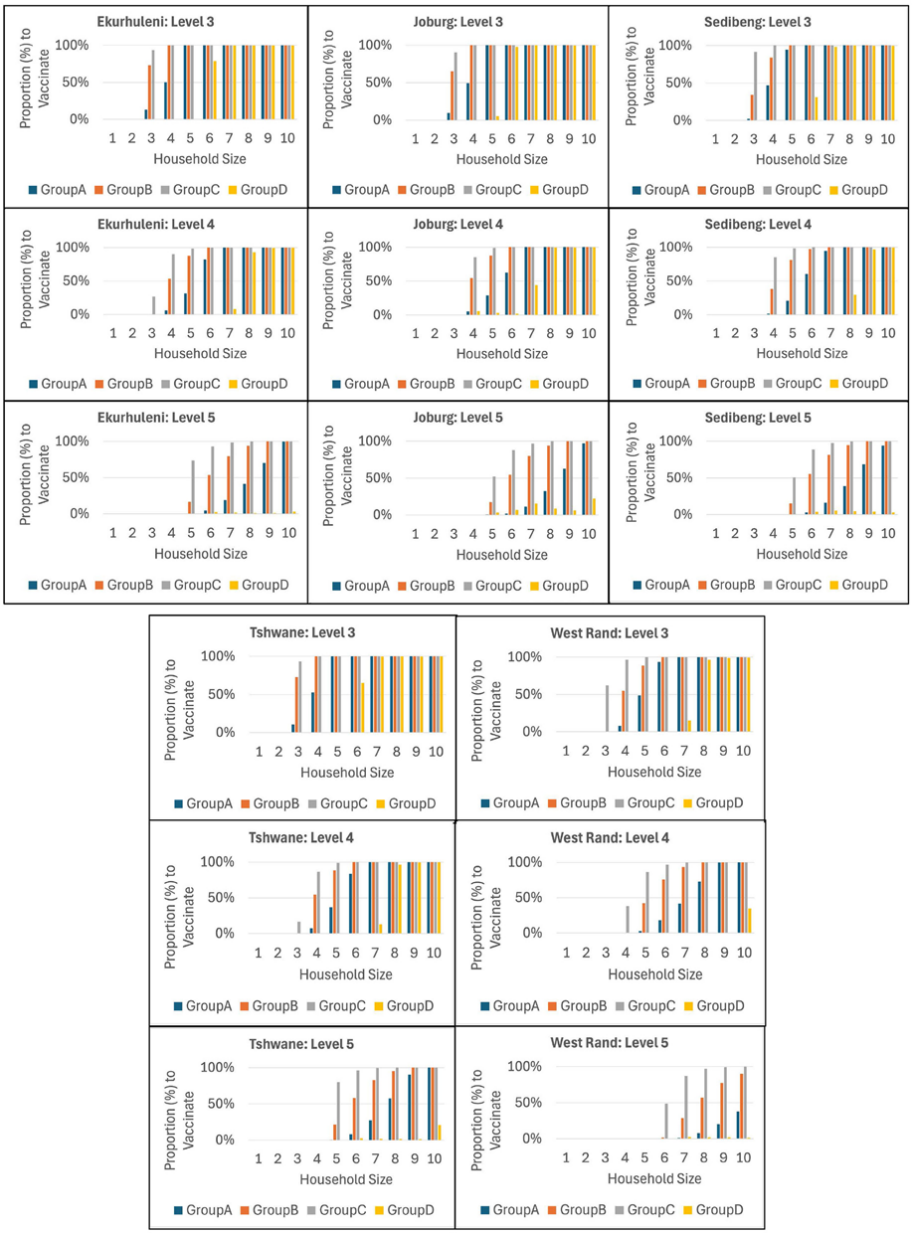
The integrated chance constraints stochastic programming (ICC-SP) model optimal proportion to vaccinate in each household size to prevent epidemics under Medium Reliability and fixed intervention level with vaccination against infection (VEI) as the vaccine efficacy criterion. The bar graphs are for each age group: Group A (1−19, blue), Group B (20−39, orange), Group C (40−64, gray), and Group D (65 & up, yellow).

**Figure 7:**
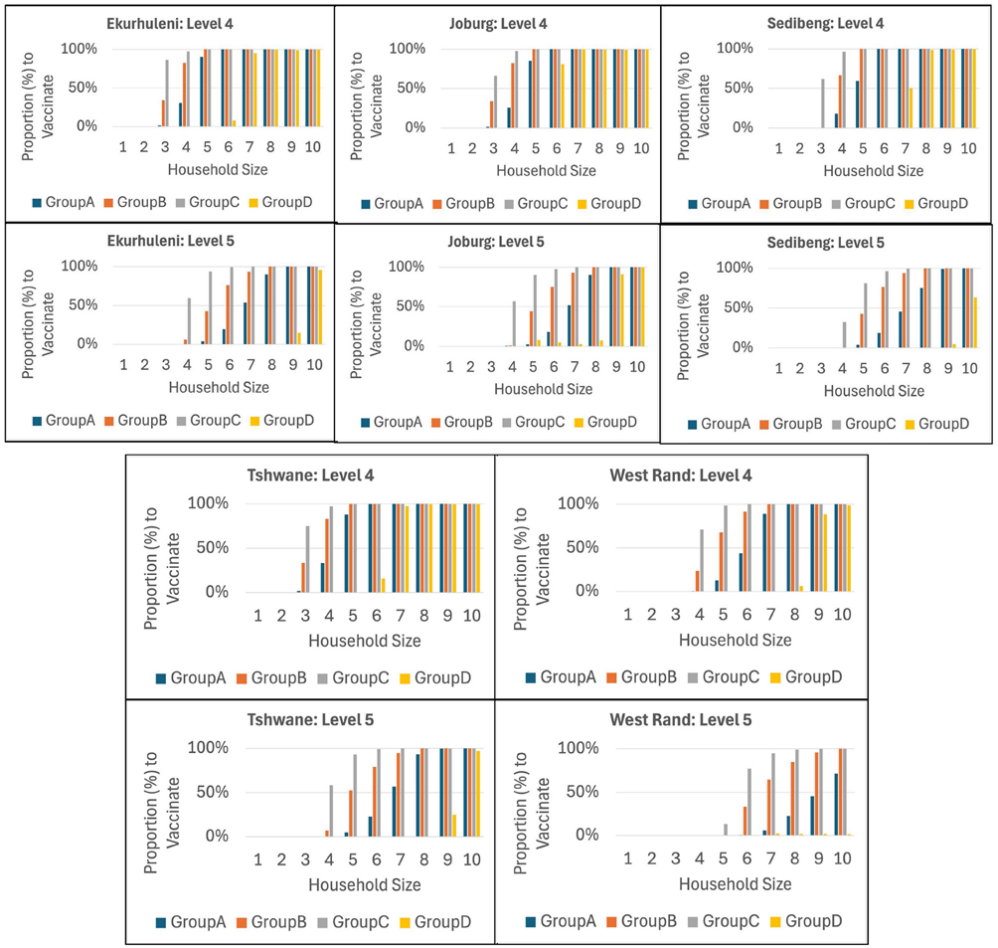
The integrated chance constraints stochastic programming (ICC-SP) model optimal proportion to vaccinate in each household size to prevent epidemics under High Reliability and fixed intervention level with vaccination against infection (VEI) as the vaccine efficacy criterion. The bar graphs are for each age group: Group A (1−19, blue), Group B (20−39, orange), Group C (40−64, gray), and Group D (65 & up, yellow).

Our ICC-SP model is a heterogeneous model in terms of age and therefore, besides determining the optimal total proportion to vaccine to prevent epidemics in each municipality, the model also computes the corresponding proportion that must be vaccinated in each age group for each household size. Recall that the age groups are *A* (*age* ≤ 19years), *B* (20 ≤ *age* ≤ 39years), *C* (40 ≤ *age* ≤ 64years), and *D* (*age*≥65years). In addition, in this case study we assume age-related differences in infectivity such that Group D (older age group) has lower relative infectivity than Groups C, B, and A (younger age group), in that order. We report the computational results using bar graphs in order to visualize the trends in the proportion to vaccinate in each age group. Figures 6, 7 show the plots for VEI as the vaccine efficacy criterion under Medium and High reliability levels, respectively. The graphs provide useful information for public health decision makers, and it is interesting to see the variations in the trends between municipalities at all levels of intervention from Level 1 to 5.

As can be seen in the figures, the proportion to vaccinate for each age group increases with household size in general at each intervention level, with Group D being prominent only in larger households. Overall, we observe that age groups *B* and *C* are prioritized with age groups *A* and *D* appearing in larger households. Clearly, larger household sizes require vaccinating more of the population in all the age groups while household sizes 1 and 2 (in some cases size 3 as well) do not require vaccinating anyone. We can observe that groups *C*, *B*, and *A* are prioritized in that order starting from smaller to larger households, while Group *D* is not prioritized in smaller households. As expected, we also see that higher intervention levels require vaccinating relatively smaller proportions of the population to prevent epidemics. Notice that the older age group *D* is the least to vaccinate at intervention Level 5 in all municipalities. In terms of reliability levels, the results show that the proportion to vaccinate increases with reliability level. Consider, for example, Ekurhuleni under intervention Level 4. To prevent epidemics, *all* age groups must be vaccinated at 100% only for households of size 10 under Low reliability; sizes 9 and 10 under Medium reliability; and sizes 8, 9, and 10 under High reliability. We also obtained age-related proportions to vaccinate to prevent epidemics using VES and VEH as vaccine efficacy criteria. The results show similar trends to VEI in general, albeit the proportions to vaccinate are relatively lower for VES and VEH, in that order. Groups *C* and *B* are prioritized, followed by *A* and then *D*. When we consider the case where Group A has lower relative infectivity than Groups *B*, *C*, and *D*, in that order (older population is more infective), the results show that Groups *C* and *B* are prioritized, followed by *D* and then *A*.

## 4 Discussion

This computational study reveals that of the three vaccine efficacy criteria, VEI is the strictest criterion to determine optimal vaccination strategies, followed by VES and then VEH. VEI considers actual infection of the disease, whether symptomatic or asymptomatic, and typically affects most of the population during an epidemic and is measured by testing. However, VES involves severe symptoms and may only affect a subset (e.g., certain age groups) of the population. Similarly, VEH involves hospitalization and tends to affect a subset of the population of symptomatic cases. However, both VES and VEH can have a significant impact on optimal vaccination strategies, especially for virus variants that cause severe symptoms that lead to hospitalization. In general, the results clearly show that a higher intervention level generally requires vaccinating a relatively smaller proportion of the population to control epidemics. We observe that under High reliability epidemics can only be controlled by imposing a relatively high intervention level in order to achieve vaccination strategies that are practically feasible. This observation confirms the need for intervention control measures coupled with vaccinations to bring epidemics under control.

The results show that vaccination allocations are different based on the population and demographics of each municipality. In particular, the results reveal that Tshwane and Ekurhuleni require the highest proportion to vaccinate under the three vaccine efficacy criteria (VEI, VES, and VEH) followed by Johannesburg, Sedibeng, and West Rand, in that order. West Rand requires the least proportion to vaccinate across all intervention levels. Recall that among the five municipalities, Johannesburg has the largest population of 5,079,469 with a population density of 3,088/km^2^. It is followed by Ekurhuleni with a population of 4,024,285 and a population density of 2,038/km^2^. Next is Tshwane with a population of 3,832,516 and population density of 609/km^2^ followed by Sedibeng with a population of 1,125,282 and a population density of 270/km^2^. West Rand has the smallest population of 1,007,757 and a population density of 247/km^2^. Apart from each municipality's overall population and population density, more granular factors that influence the optimal vaccination strategy include household characteristics such as age heterogeneity and size, other household demographics, geospatial features, and socioeconomic factors.

Prioritizing who to vaccinate in terms of age varies with relative infectivity and susceptibility of each age group. When the younger population is relatively more infective than the older population, Groups *C* and *B* are prioritized, followed by *D* and then *A*. There is a general trend to prioritize vaccinating the younger and mid-age group population (groups *B* and *C*) in smaller families, and the older age group (Group *D*) in larger households. This is true across the five municipalities, and the reason is that the younger population has higher relative infectivity compared to the older population. When the older population is more infective than the younger population, Groups *B* and *C* are prioritized, followed by *D* and then *A*. This is due to the older population having higher relative infectivity compared to the younger population. In essence, the combined relative infectivity and susceptibility of the age groups appear to be relatively high for mid-age groups, therefore prioritizing vaccinating age groups *B* and *C* to prevent epidemics.

In terms of ethical and equity considerations, prioritizing the younger population (assuming having high relative infectivity) can be effective in reducing and/or preventing epidemics, but can come at the expense of adverse effects (e.g., severity of symptoms, hospitalization, mortality) for the older population, especially if they have high relative susceptibility. On the contrary, prioritizing the older population may minimize those adverse effects, but may result in not containing the disease outbreaks due to the younger population spreading the disease. The ICC-SP model could be extended to include equity constraints in two keyways. First, one can set the relative infectivity and susceptibility parameters in the model for the different age groups appropriately to reflect the needed equity considerations. Second, vaccine constraints can be incorporated into the model to reflect how many vaccines are allocated to each age group to reflect the equity considerations at hand.

Other household demographics that can be critical include family and marital status, gender distribution, and income. How best to deal with a single-family multi-person household from one family may differ significantly from dealing with a multi-family multi-person household. Geospatial features and socioeconomic factors also come into play. Due to the geospatial legacy of South Africa's policies, certain interventions in high-density, low-income township areas may be less effective than in low-density, high-income surburban areas. It may be necessary to vary the nature of the types of interventions not only between municipalities but also within municipalities.

## 5 Conclusion

We derive a data-driven integrated chance constraints stochastic programming (ICC-SP) approach for finding optimal vaccination strategies under uncertainty to prevent epidemics in a multi-community setting. This new approach involves a stochastic optimization model of disease spread that includes multi-community population demographic data, uncertainty disease spread data (susceptibility and infectivity), and uncertain vaccine efficacy data. The model determines the optimal vaccination strategies, i.e., that is the proportion of individuals in a given community and household type to vaccinate to bring the reproduction number below one under a given decision-maker's reliability (or risk) level. This data-driven approach was implemented and tested based on COVID-19 data for five neighboring municipalities in Gauteng province, South Africa. The results reveal, among other insights, that to prevent or control epidemics, vaccination strategies should be designed to prioritize vaccinating specific households and age groups with high levels of combined relative susceptibility and infectivity. The results also show that intervention levels play a key role in the containment of epidemics. Specifically, high levels of intervention reduce the proportion of the population that has to be vaccinated. A key advantage of the ICC-SP model is that it is data-driven and its decisions adapt to the underlying stochastic data fed to the model. In addition, the model enables the decision maker to consider different levels of risk, allowing for what-if analyses for public health policy. We believe that to be ready for future epidemics, there is still a strong need for data-driven optimization models to determine optimal vaccination strategies that adapt to new virus variants and uncertain vaccine efficacy. Future work along this line of research includes incorporating vaccine hesitancy and vaccine distribution logistics into the ICC-SP model.

## Data Availability

The original contributions presented in the study are included in the article/Supplementary material, further inquiries can be directed to the corresponding author/s.
