# Supplementary material for: Optimizing vaccination strategies under uncertainty to prevent epidemics
Source: Front Public Health. 2026 Feb 24;14:1761731. doi: 10.3389/fpubh.2026.1761731 (PMC12971655; doi:10.3389/fpubh.2026.1761731)
Supplement: Supplementary file 1 [file Data_Sheet_1.pdf]

Supplementary Material

1 SUPPLEMENTARY DATA

This supplementary material includes population demographic distribution data used in the case study and disease and vaccine efficacy data. We report the two types of data in Subsections 1.1 and 1.2, respectively.

1.1 Supplementary Tables – Population Demographic Data Used in the Case Study

The population data for each municipality in Gauteng province were obtained from Statistics South Africa (2022a), while the household size distribution data for each of the municipalities were downloaded from the reports in the Census 2022 Products section of the census website for the year 2022 (Statistics South Africa, 2022b).

Age Group	Ekurhuleni	Johannesburg	Sedibeng	Tshwane	West Rand
Group A	1171192	1368854	374990	1201973	308293
Group B	1673121	2009848	427734	1568608	384711
Group C	1017241	1185261	311117	1023034	252143
Group D	205058	239146	76813	246344	53218
Total	4066612	4803109	1190654	4039960	998364

A $(age \leq 19$ years$)$, B $(20 \leq age \leq 39$ years$)$, C $(40 \leq age \leq 64$ years$)$, D $(age \geq 65$ years$)$

Table S1. Population age group data for the five municipalities used in the case study.

Age Group	Ekurhuleni	Johannesburg	Sedibeng	Tshwane	West Rand
Group A	0.288	0.285	0.315	0.297	0.309
Group B	0.411	0.418	0.359	0.388	0.385
Group C	0.250	0.247	0.261	0.253	0.253
Group D	0.050	0.050	0.065	0.061	0.053
Total	1.000	1.000	1.000	1.000	1.000

A $(age \leq 19$ years$)$, B $(20 \leq age \leq 39$ years$)$, C $(40 \leq age \leq 64$ years$)$, D $(age \geq 65$ years$)$

Table S2. Proportions of each age group in the population data for the five municipalities used in the case study.

Household Size	Ekurhuleni	Johannesburg	Sedibeng	Tshwane	West Rand	Total
1	404198	511492	90935	360141	100914	1467680
2	309440	427941	82318	282085	78356	1180140
3	305132	425270	79708	273678	76134	1159922
4	187953	238337	57823	189357	46561	720031
5	101078	116004	33174	106538	26107	382900
6	51141	56166	16251	52277	13111	188946
7	26657	28309	8166	26392	7100	96624
8	14627	15526	4072	13635	3634	51495
9	8471	8927	2117	7586	1987	29088
10	5056	5253	1079	4477	1187	17051
11	2877	3246	636	2324	664	9747
12	1641	2062	313	1493	317	5825
13	1063	1266	137	920	174	3560
14	651	733	95	525	128	2133
15	380	465	56	309	60	1270
16	242	346	34	202	26	850
17	132	169	22	129	31	484
18	87	126	15	64	12	305
19	65	87	5	41	16	214
20	37	56	8	26	2	129
21	22	51	4	23	2	101
22	25	30	2	3	2	61
23	8	19	0	5	2	33
24	8	16	0	6	0	30
25	3	6	2	4	3	18
26	3	6	0	0	0	9
27	0	3	0	3	0	6
28	2	2	0	4	0	7
29	2	0	0	0	0	2
30	3	3	2	4	0	11
31	0	0	0	0	0	0
32	0	0	0	0	0	0
33	0	0	0	0	0	0
34	0	0	0	0	0	0
45	0	0	0	0	0	0
50	0	0	0	0	0	0
Total	1421003	1841917	376971	1322252	356530	5318672

Table S3. Household data (number of households) for the five municipalities used in the case study.

Age in Completed Years	Ekurhuleni	Johannesburg	Sedibeng	Tshwane	West Rand	Total
0	74885	92388	20792	75305	19544	282914
1	68541	89500	19608	70566	16674	264890
2	68895	84430	20435	70519	17467	261747
3	65122	75701	19493	67327	17172	244815
4	62867	73608	19266	64766	16481	236988
5	52966	61602	16454	55511	14056	200588
6	53901	62639	17827	56965	14106	205438
7	54955	63765	18047	58708	14883	210358
8	54640	63130	18096	58230	14994	209090
9	55931	63334	18732	58980	14719	211695
10	56030	62885	18350	57624	14900	209789
11	58178	66194	20029	59459	15442	219301
12	57727	64864	19452	58617	14885	215546
13	59194	65807	20137	59733	15821	220691
14	55931	63519	19239	56394	15112	210195
15	56376	63498	19060	55651	15041	209626
16	55816	63104	18679	54645	14892	207136
17	54441	61812	17994	54958	14502	203707
18	52348	62614	16720	53988	13645	199315
19	52449	64461	16581	54027	13956	201474
20	66402	80814	20412	67467	17871	252966
21	75320	93233	22186	74331	19623	284693
22	74499	89857	21233	73279	18729	277598
23	75137	88557	20424	70105	18329	272553
24	77830	91863	20431	73313	18798	282235
25	84367	99971	21526	77326	19802	302991
26	85983	103584	21270	78354	19241	308431
27	88663	109002	22104	82366	20025	322160
28	88007	106697	21694	79372	19621	315391
29	94941	109484	22162	86776	20107	333470
30	89535	104014	21887	82120	19766	317323
31	97111	118991	23128	88153	21259	348642
32	91397	112160	22667	83849	20343	330416
33	87531	104832	21667	81827	19707	315565
34	84773	100987	20527	79626	18172	304084
35	87277	103621	21207	80950	19246	312301
36	88745	106415	21520	84123	20125	320928
37	79856	95567	20567	76725	17776	290492
38	76552	93608	20191	73138	17389	280879
39	79194	96590	20929	75408	18783	290905
40	67023	81404	18528	64786	15906	247647
41	74098	87575	19451	68408	16871	266402
42	66577	79292	18283	64277	15629	244059
43	59915	71131	16755	57795	14025	219621
44	56474	68025	15947	56560	14149	211155
46	49493	57925	14275	49532	11509	182735
47	47252	55228	13965	47536	11303	175284
48	42399	49838	12910	43736	10277	159160
49	44782	52680	13818	45686	10811	167777
50	36703	42984	11491	38476	8950	138604
51	41902	47935	12653	42113	10189	154793
52	37966	43237	11818	39295	9752	142068
53	36491	42155	11500	37741	9677	137564
54	31000	35778	10188	32928	8231	118125
55	30711	35035	10097	32351	7954	116147
56	32516	37099	10806	32729	8648	121798
57	30669	34687	10269	31655	8030	115310
58	30103	33570	10629	31013	8151	113466
59	29562	33092	10232	30052	8206	111144
60	24668	28246	8508	26242	6587	94250
61	27847	31251	9698	28227	7217	104241
62	23932	27529	8643	25343	6485	91932
63	24651	27097	9025	24746	6315	91834
64	21487	23949	7780	23234	5702	82151
65	20399	23423	7409	22423	5208	78862

Table S4. Population age data (1 to 65 in completed years) for the five municipalities used in the case study.

Age in Completed Years	Ekurhuleni	Johannesburg	Sedibeng	Tshwane	West Rand	Total
66	18494	20846	6872	20120	4892	71225
67	17051	19195	6435	18879	4365	65925
68	15743	18147	5892	17794	4086	61662
69	16216	18711	6269	17662	3986	62844
70	11602	13109	4270	13236	3073	45290
71	13160	15015	5216	14181	3430	51002
72	10802	12619	4111	12567	2659	42758
73	10208	11638	3678	12224	2745	40494
74	9128	10599	3560	11327	2315	36928
75	7999	9198	3067	10001	2138	32403
76	7542	8471	2737	9413	1926	30090
77	6271	7018	2299	8054	1621	25263
78	5545	6561	2056	7515	1440	23116
79	5634	6736	2170	7437	1409	23387
80	4299	5071	1473	5914	1098	17855
81	4460	5430	1676	6020	1205	18792
82	3467	4359	1371	5205	882	15284
83	2970	3901	1045	4470	808	13194
84	2486	3237	925	3669	762	11079
85	2125	2954	842	3482	674	10078
86	1802	2210	663	2724	490	7889
87	1482	1907	522	2215	370	6496
88	1138	1639	564	1792	340	5472
89	1018	1487	347	1671	286	4809
90	786	1061	256	1148	202	3452
91	750	1068	298	1204	172	3493
92	500	793	192	874	119	2477
93	410	550	123	669	96	1847
94	271	428	89	574	85	1447
95	196	296	56	333	50	932
96	189	220	63	253	34	759
97	113	192	44	211	43	602
98	78	153	14	149	26	421
99	428	538	118	432	116	1631
100	46	52	9	102	14	224
101	51	98	21	128	12	310
102	39	50	20	58	8	175
103	25	31	16	56	6	134
104	26	10	6	27	5	74
105	11	16	0	12	3	42
106	6	8	1	13	6	34
107	16	10	3	26	2	57
108	4	8	0	12	0	24
109	12	6	4	5	3	30
110	2	9	0	5	0	16
111	6	4	0	13	0	23
112	3	3	0	2	0	8
113	3	6	2	9	2	21
114	6	3	0	5	0	15
115	0	6	0	7	0	13
116	2	8	2	3	0	15
117	6	3	0	3	0	12
118	4	2	0	0	2	8
119	5	5	0	3	2	14
120	0	0	0	3	2	5
121	9	9	0	4	0	22
122	0	2	0	4	0	6
123	0	0	0	0	0	0
124	3	6	0	0	0	9
125	5	2	3	2	0	11
126	0	0	0	2	0	2
127	3	4	0	0	0	8
128	0	0	0	0	2	2
129	2	3	5	0	0	9
130	0	2	0	0	2	4
Unspecified	79	153	34	355	102	723
Total	4066691	4803262	1190688	4040315	998466	15099423

Table S5. Population age data (66 to 130 in completed years) for the five municipalities used in the case study.

1.2 Supplementary Tables – Disease and Vaccine Data Used in the Case Study

The probability distribution data for inside contact rate model parameter $b(\tilde{\omega})$ were generated based on Bhatt et al. (2022) and Jing et al. (2020), while that for the outside contact rate model parameter $m(\tilde{\omega})$ were generated based on historical time series data for the effective reproduction number R_t from NICD (2021) and effective contact rates from Mukandavire et al. (2020). For vaccine efficacy model parameter $\epsilon(\tilde{\omega})$, the Probability distribution data for vaccine effectiveness against infection (VEI), symptomatic cases (VES), and hospitalization (VEH) where generated based on the J&J and Pfizer vaccines (Department of Health Republic of South Africa, 2021; Terry, 2021; Ssentongo et al., 2022; WHO, 2021). The probability distributions for relative susceptibility model parameter $\beta(\tilde{\omega})$ were generated based on the results in Jassat et al. (2022) Russell et al. (2023), while the distributions for relative infectivity model parameter $\lambda(\tilde{\omega})$ were based on several studies (Prem et al., 2017; Goldstein et al., 2020; Reid, 2021).

**Probability Distribution for Inside Transmission Rate**				
Probability	0.1	0.4	0.4	0.1
Transmission Rate	0.4	0.3	0.2	0.1

Table S6. Probability distribution for within household transmission rate $b(\tilde{\omega})$ for the five municipalities used in the case study.

**Probability Distribution for Outside Transmission Rate $m(\tilde{\omega})$ for Ekurhuleni**						
Level 1						
Probability	0.171	0.317	0.220	0.146	0.098	0.049
Transmission Rate	2.396	2.171	1.765	1.348	0.917	0.657
Level 2						
Probability	0.171	0.317	0.220	0.146	0.098	0.049
Transmission Rate	2.300	2.080	1.688	1.287	0.873	0.624
Level 3						
Probability	0.171	0.317	0.220	0.146	0.098	0.049
Transmission Rate	2.108	1.899	1.535	1.164	0.786	0.558
Level 4						
Probability	0.171	0.317	0.220	0.146	0.098	0.049
Transmission Rate	1.773	1.583	1.266	0.950	0.633	0.443
Level 5						
Probability	0.171	0.317	0.220	0.146	0.098	0.049
Transmission Rate	1.389	1.221	0.959	0.705	0.458	0.312

Table S7. Probability distribution for outside household transmission rate $m(\tilde{\omega})$ for Ekurhuleni used in the case study.

Probability Distribution for Outside Transmission Rate $m(\tilde{\omega})$ for Johannesburg						
Level 1						
Probability	0.122	0.268	0.268	0.195	0.098	0.049
Transmission Rate	2.393	2.169	1.763	1.347	0.916	0.656
Level 2						
Probability	0.122	0.268	0.268	0.195	0.098	0.049
Transmission Rate	2.298	2.078	1.687	1.286	0.872	0.623
Level 3						
Probability	0.122	0.268	0.268	0.195	0.098	0.049
Transmission Rate	2.106	1.898	1.533	1.163	0.785	0.558
Level 4						
Probability	0.122	0.268	0.268	0.195	0.098	0.049
Transmission Rate	1.771	1.581	1.265	0.949	0.633	0.443
Level 5						
Probability	0.122	0.268	0.268	0.195	0.098	0.049
Transmission Rate	1.388	1.220	0.958	0.704	0.458	0.312

Table S8. Probability distribution for outside household transmission rate $m(\tilde{\omega})$ for Johannesburg used in the case study.

Probability Distribution for Outside Transmission Rate $m(\tilde{\omega})$ for Sedibeng						
Level 1						
Probability	0.137	0.176	0.353	0.196	0.078	0.059
Transmission Rate	2.351	2.131	1.733	1.323	0.900	0.644
Level 2						
Probability	0.137	0.176	0.353	0.196	0.078	0.059
Transmission Rate	2.257	2.042	1.657	1.263	0.857	0.777
Level 3						
Probability	0.137	0.176	0.353	0.196	0.078	0.059
Transmission Rate	2.069	1.864	1.507	1.143	0.771	0.548
Level 4						
Probability	0.137	0.176	0.353	0.196	0.078	0.059
Transmission Rate	1.740	1.554	1.243	0.932	0.621	0.435
Level 5						
Probability	0.137	0.176	0.353	0.196	0.078	0.059
Transmission Rate	1.364	1.199	0.942	0.692	0.450	0.306

Table S9. Probability distribution for outside household transmission rate $m(\tilde{\omega})$ for Sedibeng used in the case study.

Probability Distribution for Outside Transmission Rate $m(\tilde{\omega})$ for Tshwane						
Level 1						
Probability	0.220	0.244	0.244	0.171	0.049	0.073
Transmission Rate	2.366	2.192	1.783	1.361	0.926	0.663
Level 2						
Probability	0.220	0.244	0.244	0.171	0.049	0.073
Transmission Rate	2.271	2.101	1.705	1.300	0.882	0.630
Level 3						
Probability	0.220	0.244	0.244	0.171	0.049	0.073
Transmission Rate	2.082	1.918	1.550	1.176	0.794	0.564
Level 4						
Probability	0.220	0.244	0.244	0.171	0.049	0.073
Transmission Rate	1.751	1.599	1.279	0.959	0.639	0.448
Level 5						
Probability	0.220	0.244	0.244	0.171	0.049	0.073
Transmission Rate	1.372	1.233	0.969	0.712	0.463	0.315

Table S10. Probability distribution for outside household transmission rate $m(\tilde{\omega})$ for Tshwane used in the case study.

**Probability Distribution for Outside Transmission Rate $m(\tilde{\omega})$ for West Rand**						
Level 1						
Probability	0.123	0.175	0.281	0.246	0.140	0.035
Transmission Rate	1.893	1.716	1.395	1.065	0.725	0.519
Level 2						
Probability	0.123	0.175	0.281	0.246	0.140	0.035
Transmission Rate	1.818	1.644	1.334	1.017	0.690	0.620
Level 3						
Probability	0.123	0.175	0.281	0.246	0.140	0.035
Transmission Rate	1.666	1.501	1.213	0.920	0.621	0.441
Level 4						
Probability	0.123	0.175	0.281	0.246	0.140	0.035
Transmission Rate	1.401	1.251	1.001	0.751	0.500	0.350
Level 5						
Probability	0.123	0.175	0.281	0.246	0.140	0.035
Transmission Rate	1.098	0.965	0.758	0.557	0.362	0.246

Table S11. Probability distribution for outside household transmission rate $m(\tilde{\omega})$ for West Rand used in the case study.

**Probability Distribution for Vaccine Efficacy $\epsilon(\tilde{\omega})$**			
Probability	0.25	0.50	0.25
VEI	0.40	0.50	0.60
VES	0.60	0.65	0.70
VEH	0.80	0.95	0.85

VEI: Vaccine efficacy against infection. VES: Vaccine efficacy against severe symptoms. VEH: Vaccine efficacy against hospitalization.

Table S12. Probability distribution for vaccine efficacy $\epsilon(\omega)$ for the five municipalities in used in the case study.

Probability Distribution for Relative Susceptibility $\beta(\tilde{\omega})$ for Ekurhuleni

Probability	Group A	Group B	Group C	Group D
0.50	0.60	1.00	1.30	1.70
0.50	0.80	1.00	1.15	1.35
Proportion in population	0.29	0.39	0.27	0.05

Table S13. Probability distribution for relative susceptibility $\beta(\tilde{\omega})$ for Ekurhuleni used in the case study.

Probability Distribution for Relative Susceptibility $\beta(\tilde{\omega})$ for Johannesburg

Probability	Group A	Group B	Group C	Group D
0.50	0.60	1.00	1.30	1.76
0.50	0.80	1.00	1.15	1.38
Proportion in population	0.29	0.40	0.26	0.05

Table S14. Probability distribution for relative susceptibility $\beta(\tilde{\omega})$ for Johannesburg used in the case study.

Probability Distribution for Relative Susceptibility $\beta(\tilde{\omega})$ for Sedibeng

Probability	Group A	Group B	Group C	Group D
0.50	0.60	1.00	1.30	1.60
0.50	0.80	1.00	1.15	1.30
Proportion in population	0.30	0.35	0.30	0.05

Table S15. Probability distribution for relative susceptibility $\beta(\tilde{\omega})$ for Sedibeng used in the case study.

Probability Distribution for Relative Susceptibility $\beta(\tilde{\omega})$ for Tshwane				
Probability	Group A	Group B	Group C	Group D
0.50	0.60	1.00	1.30	1.84
0.50	0.80	1.00	1.15	1.42
Proportion in population	0.30	0.39	0.26	0.05

Table S16. Probability distribution for relative susceptibility $\beta(\tilde{\omega})$ for Tshwane used in the case study.

Probability Distribution for Relative Susceptibility $\beta(\tilde{\omega})$ for West Rand				
Probability	Group A	Group B	Group C	Group D
0.50	0.60	1.00	1.30	1.60
0.50	0.80	1.00	1.15	1.30
Proportion in population	0.30	0.35	0.30	0.05

Table S17. Probability distribution for relative susceptibility $\beta(\tilde{\omega})$ for West Rand used in the case study.

Probability Distribution for Relative Infectivity $\lambda(\tilde{\omega})$ for Ekurhuleni				
Probability	Group A	Group B	Group C	Group D
0.50	1.30	1.00	0.80	0.34
0.50	1.20	1.00	0.90	0.38
Proportion in population	0.29	0.39	0.27	0.05

Table S18. Probability distribution for relative infectivity $\lambda(\tilde{\omega})$ for Ekurhuleni used in the case study.

Probability Distribution for Relative Infectivity $\lambda(\tilde{\omega})$ for Johannesburg				
Probability	Group A	Group B	Group C	Group D
0.50	1.30	1.00	0.80	0.30
0.50	1.20	1.00	0.90	0.36
Proportion in population	0.29	0.40	0.26	0.05

Table S19. Probability distribution for relative infectivity $\lambda(\tilde{\omega})$ for Johannesburg used in the case study.

Probability Distribution for Relative Infectivity $\lambda(\tilde{\omega})$ for Sedibeng				
Probability	Group A	Group B	Group C	Group D
0.50	1.30	1.00	0.80	0.30
0.50	1.20	1.00	0.90	0.38
Proportion in population	0.32	0.35	0.27	0.06

Table S20. Probability distribution for relative infectivity $\lambda(\tilde{\omega})$ for Sedibeng used in the case study.

Probability Distribution for Relative Infectivity $\lambda(\tilde{\omega})$ for Tshwane				
Probability	Group A	Group B	Group C	Group D
0.50	1.30	1.00	0.78	0.34
0.50	1.20	1.00	0.88	0.42
Proportion in population	0.30	0.39	0.26	0.05

Table S21. Probability distribution for relative infectivity $\lambda(\tilde{\omega})$ for Tshwane used in the case study.

Probability Distribution for Relative Infectivity $\lambda(\tilde{\omega})$ for West Rand				
Probability	Group A	Group B	Group C	Group D
0.50	1.30	1.00	0.81	0.34
0.50	1.20	1.00	0.90	0.40
Proportion in population	0.30	0.35	0.30	0.05

Table S22. Probability distribution for relative infectivity $\lambda(\tilde{\omega})$ for West Rand used in the case study.

REFERENCES

[Dataset] Statistics South Africa. SuperWEB2 (2022a). https://superweb.statssa.gov.za/webapi/jsf/tableView/tableVie[illegible] Accessed: 2024-05-01.

[Dataset] Statistics South Africa. Official Stats SA Census Portal (2022b). https://census.statssa.gov.za/#/.Accessed: 2024-05-01.

Bhatt M, Plint AC, Tang K, Malley R, Huy AP, McGahern C, et al. Household transmission of SARS-CoV-2 from unvaccinated asymptomatic and symptomatic household members with confirmed SARS-CoV-2 infection: an antibody-surveillance study. *Canadian Medical Association Open Access Journal* **10** (2022) E357–E366. doi:10.9778/cmajo.20220026.

Jing QL, Liu MJ, Zhang ZB, Fang LQ, Yuan J, Zhang AR, et al. Household secondary attack rate of COVID-19 and associated determinants in Guangzhou, China: a retrospective cohort study. *The Lancet Infectious Diseases* **20** (2020) 1141–1150.

[Dataset] NICD. The initial and daily covid-19 effective reproductive number in south africa (2021). https://www.nicd.ac.za/diseases-a-z-index/disease-index-covid-19/surveillance-reports/covid-19-special-reports/the-initial-and-daily-covid-19-effective-reproductive-number-in-south-africa/. Accessed: 2022-04-11.

Mukandavire Z, Nyabadza F, Malunguza NJ, Cuadros DF, Shiri T, Musuka G. Quantifying early COVID-19 outbreak transmission in south africa and exploring vaccine efficacy scenarios plos one. *PLOS ONE* **15** (2020). E0236003.

[Dataset] Department of Health Republic of South Africa. Media Statement: South Africa commences early access vaccine rollout to Healthcare Workers - Sisonke! Let's work together to protect our Healthcare Workers (2021). https://www.health.gov.za/wp-content/uploads/2021/02/Media-Statement-South-Africa-rollout-Covid-19-Vaccine.pdf. Accessed: 2024-06-01.

[Dataset] Terry M. With Evidence of Efficacy, South Africa to Test J&J Vaccine on Health Care Workers (2021). https://www.biospace.com/article/j-and-j-s-one-shot-covid-19-vaccine-update/. Accessed: 2024-06-30.

Ssentongo P, Ssentongo A, Voleti Nea. SARS-CoV-2 vaccine effectiveness against infection, symptomatic and severe COVID-19: a systematic review and meta-analysis. *BMC Infectious Diseases* **22** (2022).

[Dataset] WHO. Vaccine efficacy, effectiveness and protection (2021). https://www.who.int/news-room/feature-stories/detail/vaccine-efficacy-effectiveness-and-protection. Accessed: 2024-06-01.

Jassat W, Mudara C, Vika C, Dryden M, Masha M, Arendse T, et al. Undiagnosed comorbidities among individuals hospitalised with COVID-19 in South African public hospitals. *South African Medical Journal* **112** (2022) 747–752.

Russell C, Lone N, Baillie J. Comorbidities, multimorbidity and COVID-19. *Nature Medicine* **29** (2023) 334–343.

Prem K, Cook AR, Jit M. Projecting social contact matrices in 152 countries using contact surveys and demographic data. *PLOS Computational Biology* **13** (2017). doi:10.1371/journal.pcbi.1005697.

Goldstein E, Lipsitch M, Cevik M. On the effect of age on the transmission of sars-cov-2 in households, schools, and the community. *The Journal of Infectious Diseases* (2020). doi:10.1093/infdis/jiaa691.

Reid T. Capturing the complexity of COVID-19 in sub-saharan africa. *Nature Africa* (2021).
